# Engineering a modular pectin-to-lipids bioconversion system using two *Kluyveromyces marxianus* strains

**DOI:** 10.1186/s40643-025-00927-z

**Published:** 2025-08-12

**Authors:** Bofan Yu, He Qiao, Xuye Lang

**Affiliations:** 1 College of Chemical and Biological Engineering, Zhejiang University, Hangzhou, 310027 China; 2 Zhejiang Key Laboratory of Intelligent Manufacturing for Functional Chemicals, ZJU-Hangzhou Global Scientific and Technological Innovation Center, Zhejiang University, Hangzhou, 311215 China; 3 Zhejiang Synthetic Biomanufacturing Innovation Center, Hangzhou, 311215 China

**Keywords:** *Kluyveromyces marxianus*, Pectin utilization, Lipids production, Modular system, Metabolic engineering

## Abstract

Pectin production by processing renewable feedstocks is an appealing and sustainable approach. However, microbial valorization of pectin remains underdeveloped and necessitates efficient expression of key enzymes. In this study, we developed a novel two-stage microbial consortium for pectin valorization through modular utilization of engineered *Kluyveromyces marxianus* strains. CRISPR-mediated integration of *PGU1* in strain YKM1013 significantly increased endoPG production, enabling efficient pectin hydrolysis and achieving a 65% improvement in hydrolysis degree. Meanwhile, strain YKM1015, engineered with a synthetic D-galacturonic acid (D-galUA) metabolic pathway, demonstrated efficient utilization of available D-galUA components. The established bioprocess achieved remarkable lipids productivity, yielding 19-fold and 6-fold increases in medium-chain fatty acid (MCFA) and long-chain fatty acid (LCFA) production, respectively, thereby establishing the microbial platform for direct pectin-to-lipids bioconversion. Collectively, these advances provided a feasible bioconversion route for valorizing pectin-rich biomass into high-value chemicals via microbial cell factory platform construction.

**Graphical Abstract:**

**Figure:**
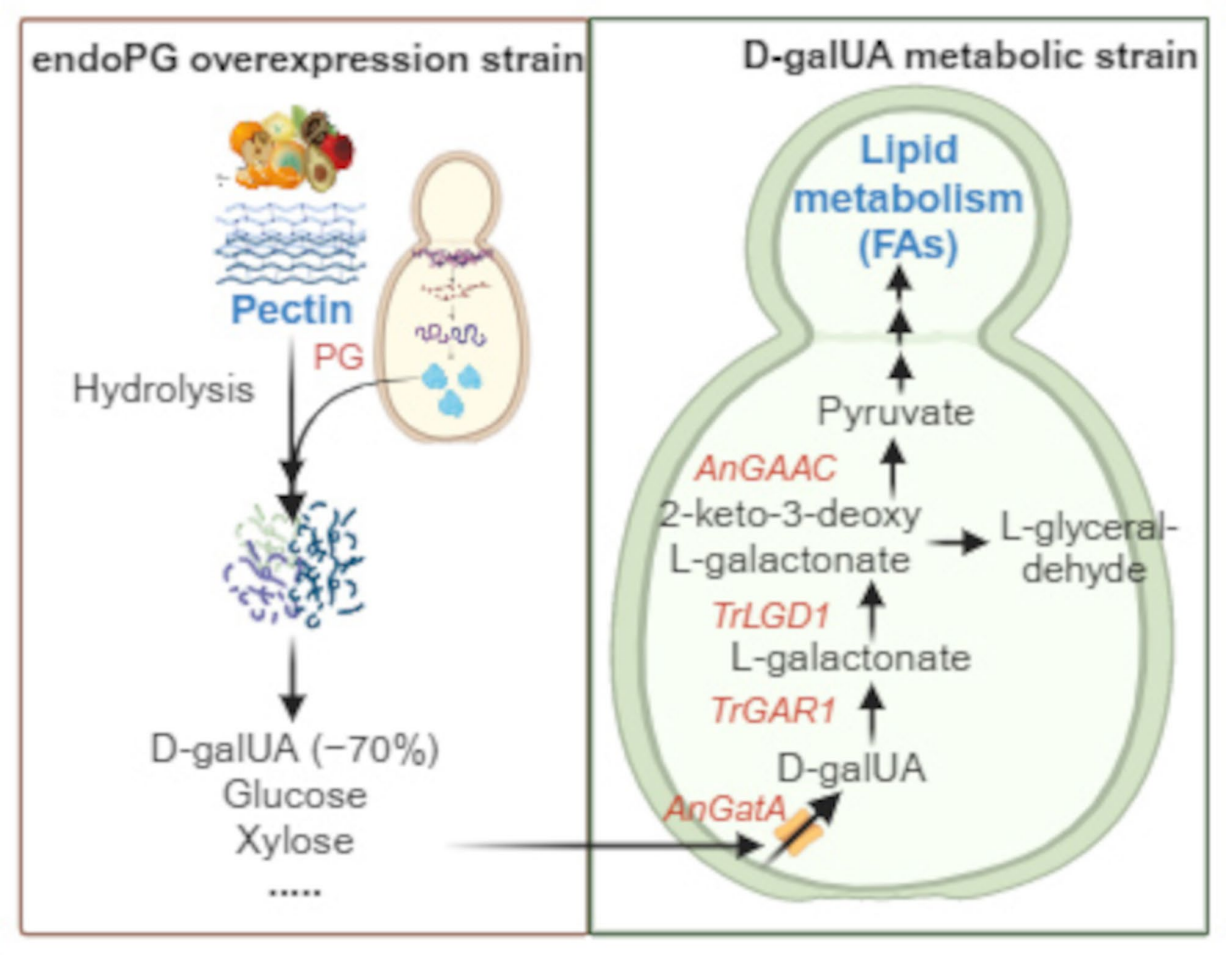

**Supplementary Information:**

The online version contains supplementary material available at 10.1186/s40643-025-00927-z.

## Introduction

Pectin is a ubiquitous polysaccharide found in the cell wall and inner layer of fruits, vegetables, and other higher plants (Xiang et al. 2024). The majority of pectin is derived from raw materials, such as fruit peels and pomaces (Williams et al. 2019; Erian and Sauer 2022). However, nearly 50% of those is regarded as waste and discarded, resulting in significant environmental pollution and unsustainable development (Chavan et al. 2019; Sharma et al. 2022). Following enzymatic depolymerization, pectin is converted to D-galUA (~ 70%) and available neutral sugars (including xylose, galactose, mannose, fucose, or arabinose) (Mohnen 2008), which can be utilized to yield valuable products. Thus, the development of a green bioconversion route is critical for the sustainable utilization of pectin. Microorganisms engineered through metabolic engineering can serve as effective cell factories offering potential to advance agricultural by-product waste treatment and generate a variety of valuable products from biomass resources (Protzko et al. 2018; Wikandari et al. 2022; Gao et al. 2023; Li et al. 2023; Zhang et al. 2024). In recent years, it has been demonstrated that the bioconversion of D-galUA components into high-value products, such as *meso*-galactaric acid and ethanol, is a viable route for pectin bioconversion, contributing to sustainable valorization and waste utilization, facilitating a circular economy (Protzko et al. 2018; Sharma et al. 2022). However, pectin bioconversion and utilization has been hindered by a lack of complete bioconversion routes and an efficient expression of key enzymes.

There has been an increased focus on acquiring enzymes for pectin metabolization that exhibit high stability and efficiency, notably the polygalacturonase (PG), pectin methylesterase (PME), and pectin lyase (PL) produced by fungi (Bonnin et al. 2014; Liang et al. 2015; Ma et al. 2018; Li et al. 2024). Besides pectin degradation, the critical enzymes associated with native D-galUA metabolism has been reported in Filamentous fungi, involving D-galacturonate reductase (GAR1) and L-galactonate dehydratase (LGD1) from *Trichoderma reesei*, and 2-keto-3-deoxy-L-galactonate aldolase (GAAC) from *Aspergillus niger* (Motter et al. 2014; Protzko et al. 2018). Nevertheless, in most yeasts, the D-galUA components are difficult to utilize due to the lack of essential enzymes that catalyze D-galUA (Grohmann et al. 1994; Huisjes et al. 2012; Biz et al. 2016). To tackle this problem, several attempts have been made by employing *Saccharomyces cerevisiae* as a production host to utilize D-galUA as a carbon source via the heterologous incorporation of D-galUA metabolic pathway, aiming to relieve pressure of pectin-rich waste on environment (Huisjes et al. 2012; Biz et al. 2016; Protzko et al. 2018; Sharma et al. 2022). Identification of GatA as a powerful D-galUA transporter derived from *A. niger* has significantly enhanced the production of *meso*-galactaric acid via a co-fermentation of D-galUA and D-glucose (Protzko et al. 2018). Also, it stated an increase of ethanol production using an engineered *S. cerevisiae* strain in the co-utilization of D-galUA and glycerol (Perpelea et al. 2022). Despite the numerous studies of D-galUA metabolism and utilization, a co-fermentation with other carbon sources was invariably required. Moreover, the lack of a comprehensive system for pectin metabolism, including pectin degradation and D-galUA metabolism, has hindered the direct utilization of pectin as a carbon source for an efficient bioconversion.

*Kluyveromyces marxianus*, an unconventional yeast strain, exhibits thermotolerance and rapid growth, utilizing a wide range of carbon sources, including C5, C6, and C12 compounds (Löbs et al. 2016; Dekker et al. 2021; Li et al. 2021). In addition, *K. marxianus* is edible and is a “generally recognized as safe” (GRAS) strain, representing a viable and attractive host for commercialization and metabolic engineering applications. Unlike conventional *S. cerevisiae*, the majority of *K. marxianus* strains are aerobic and less tolerant to alcohol fermentation, which can be exploited in biosynthesis applications to avoid by-product generation (Wagner and Alper 2016; Bilal et al. 2022). More importantly, numerous reports have demonstrated that *K. marxianus* is highly effective in secreting endo-polygalacturonase (endoPG) (Siekstele et al. 1999; Sieiro et al. 2009), which can be employed in the hydrolysis of pectin, bridging the current gap in the complete pectin metabolic process. Previous efforts to evaluate the endoPG capacity have established the critical role of *K. marxianus* in pectin hydrolysis (Wu et al. 2000; Masoud and Jespersen 2006). The production of endoPG in *K. marxianus* Y885 has improved its hydrolytic activity, boosting cell wall degradation and increasing ethanol and glycerol yields by 14% in grape pomace fermentation (Williams et al. 2019). Therefore, the reported works have established *K. marxianus* as an essential component in an optimized strategy to combine pectin degradation and D-galUA metabolism, and deliver a complete system for the bioconversion of pectin-rich biomass.

**Fig. 1:**
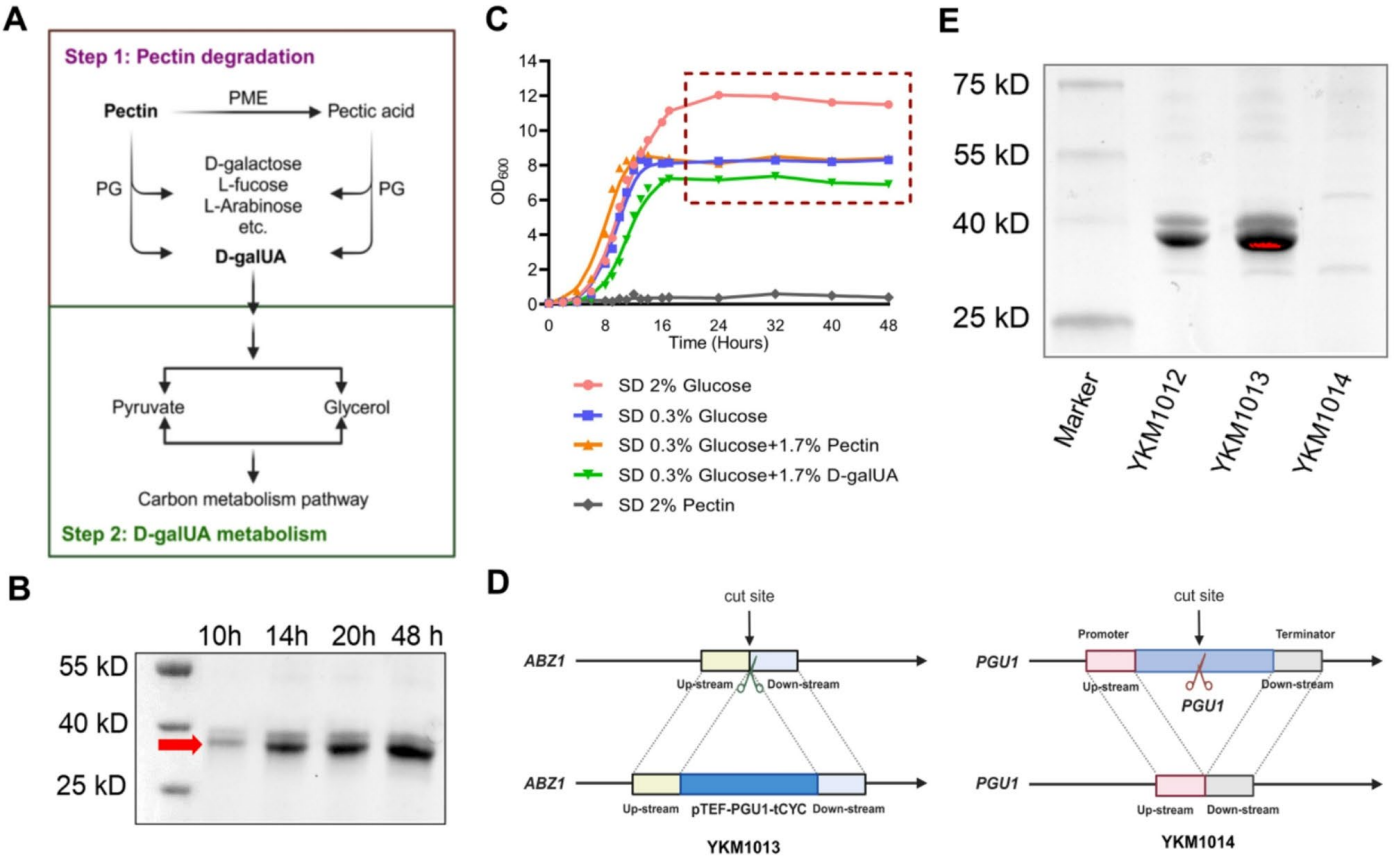
Engineering *K. marxianus* for the over-production of endoPG. (**A**) A simplified diagram of pectin metabolic pathway involves pectin degradation (step 1) and D-galUA metabolism (step 2). (**B**) The secreted endoPG of YKM1012 staining by coomassie brilliant blue (CBB) was pointed by *red arrows* by the cultivation in SD medium at 30 ℃ during different growth periods. (**C**) The growth dynamics of YKM1012 under varied carbon source compositions. The *box* with dashed line delineated the stationary-phase growth dynamics. (**D**) The schematic diagrams of construction of engineered strains YKM1013 (*left*) and YKM1014 (*right*). (**E**) The amount of endoPG secretion of different *K. marxianus* strains cultured in SD medium at 30 ℃ for 20 h staining by CBB

In this study, we developed a modular two-step system for pectin utilization (Fig. 1A). The two-step system comprised (i) pectin degradation by the endoPG-overexpressing strain YKM1013, followed by (ii) utilization of D-galUA components in *K. marxianus* YKM1015, engineered with the heterologous pathway and dedicated transporter. This constructed system was able to boost pectin utilization for cell growth, improving fatty acids (FAs) production. Together, we have successfully established an efficient bioconversion route for pectin, which promotes FAs biosynthesis and enables sustainable valorization of pectin.

## Materials and methods

### Strains, culture media and condition

All the strains used in this study were given in Table 1. The yeast cells were cultivated in either synthetic defined (SD) or yeast-extract peptone dextrose (YPD) medium. The SD medium contained 1.7 g/L Yeast Nitrogen Base without Amino Acids (BD Difco™), 1.31 g/L Yeast Amino Acids Supplement (Coolaber), and 20 g/L glucose (Coolaber). The SD-H, SD-U, and SD-H-U medium were also used, where 1.29 g/L Dropout (DO) Supplement-His (Coolaber), 1.29 g/L DO Supplement-Ura (Coolaber) and 1.27 g/L DO Supplement-His/-Ura (Coolaber) respectively replaced the Yeast Amino Acids Supplement component in the SD medium. The YPD medium was composed of 20 g/L peptone (Oxoid), 10 g/L yeast extract (Oxoid), and 20 g/L glucose (Coolaber). The yeast cells were cultivated in 250 mL flasks containing 25 mL medium with an initial optical density (OD) of 0.05. The temperature and shaker speed for yeast cell culture was 30 ℃ and 200 rpm, respectively.

**Table 1:** The summary of strains used in this study

Strains	Strains description	Source
*E. Coli* DH5α	F^−^φ80(*lacZ*)ΔM15Δ(*lacZYA-argF*)U169*end*A1*rec*A1*hsd*R17(*m*_*k*_^*−*^,*m*_*k*_^*+*^)supE44λ*-thi*-1 *gyr*A96 *rel*A1 *pho*A	TSINGKE
*E. Coli* Stbl3	F^−^ *mc*rB *mrr hsd*S20(r_B_^−^, m_B_^−^) *rec*A13 *sup*E44 *ara*-14 *gal*K2 *lac*Y1 *pro*A2*rps*L20(Str^R^) *xyl*-5λ^−^ *leu mtl*-1 endA1^+^	Thermo Fisher
YKM1012	*Kluyveromyces marxianus* CBS6556 ∆his3 ∆ura3	Obtained from Dr. Ian Wheeldon laboratory (UC Riverside)
YKM1013	YKM1012 *abz1*:: (P_*KmTEF3*_)PGU1	This study
YKM1014	YKM1012 ∆ PGU1	This study
YKM1015	YKM1012 *xyl2*:: (P_*KmGPD*_)TrGAR1 *abz1*:: (P_*KmGPD*_)TrLGD1 *lys1*:: (P_*KmGPD*_)AnGAAC *sdl1*:: (P_*KmGPD*_)AnGatA	This study
YKM1016	YKM1012 *sdl1*:: (P_*KmGPD*_)TrGAR1 *abz1*:: (P_*KmGPD*_)V-TrLGD1 *lys1*:: (P_*KmGPD*_)AnGAAC *xyl2*:: (P_*KmGPD*_)AnGatA	This study

### Plasmids construction

All the plasmids used in this study were constructed by homologous recombination and were given in Table S1. All the gene sequences and used primers were listed in Table S2 and Table S3, respectively. In this study, the plasmids were constructed through the Gibson assembly method and reassembled by using a 60-base pair insertion fragment containing 20 base pairs of upstream and downstream homologous sequences and 20 base pairs of the target sequence. In detail, the CRISPR-related plasmid was constructed by linearizing pIW601 at the PspXI (NEB) site, and the homology donor plasmids for gene integration were constructed by linearizing at XmaI and XhoI (NEB). Furthermore, the vector for PGko-HD construction was linearized by SacII and EagI (NEB). The DNA gene fragments were synthesized or amplified by polymerase chain reaction (PCR) using KOD One TM PCR Master Mix (TOYOBO). The vectors for plasmids construction were universally linearized at 37 ℃ for 1 h. After connecting the linearized vector and targeted gene fragment, the resulting plasmids containing the homologous fragment and CRISPR/Cas9 fragment were individually transformed into *Escherichia coli* (*E. coli*) *DH5α* and *Stbl3* competent cells, respectively. The transformed cells were then cultivated on Luria–Bertani (LB) agar supplemented with 100 µg/mL ampicillin at 37 ℃ for 16 h. A randomly chosen single colony was genotyped using a 2×Taq Enzyme Mix (Vazyme), and the colonies with the correct insertion size were then confirmed by Sanger sequencing for plasmids generation.

### *K. marxianus* strains construction

The transformation method has been described in a previous report (Yuan et al. 2024). The YKM1012 strain was used for transformation and strain construction. Briefly, the starting strain was cultivated in YPD mL at 30 ℃ overnight. 1 mL of the harboring cell mixture was collected by centrifugation at 5000 g for 1 min, and underwent the washing process by respectively using sterile water and 1×TE (10 mM Tris-HCl with 1 mM EDTA). After adding 500 µL transformation buffer [containing 40% polyethylene glycol 3350 (PEG), 100 mM lithium acetate (LiAc), 1×TE buffer), and 10 mM DTT], plasmids, and 10 µL of 10 mg/mL carrier DNA, the cell culture was gently mixed and then put it on ice for 10 min. Subsequently, the mixture was incubated at 47 ℃ for 15 min and centrifuged at 5000 g for 2 min. After transformation, the cell mixture was reinoculated in fresh selective media for overnight culture at 30 ℃, and then plated on the YPD agar plate for single colony confirmation by PCR amplification and sequencing. The plasmids containing the URA3 selection marker were eliminated by treating them with 1 g/L 5-fluoroorotic acid (5’-FOA) in liquid YPD medium at 30 ℃ overnight. The colonies were reinoculated on the YPD, SD-H, and SD-U agar plates to ensure the successful removal of plasmids, and stored at -80 ℃.

### Quantification of endoPG enzymatic activity

The assay for endoPG enzymatic activity was conducted by applying the 3,5-dinitrosalicylic acid (DNS) method with some minor adjustments (Miller 1959). For each sample, the endoPG secreted from the strains was used to degrade pectin in an enzymatic reaction and the mixture was incubated at 45 ℃. The thermal stability of endoPG was assessed in the 30–90 ℃ temperature range. The same volume of pectin hydrolysate and DNS were mixtured and treated in a boiling water bath for 10 min, with data collection on a microplate reader at 540 nm. Different concentrations of D-galUAs (g/L) were processed to generate the calibration curve, with an associated R^2^ > 0.999. The enzymatic activity of endoPG was assessed by measuring the reducing sugar (g/L), which was calculated according to the calibration curve. To further assess endoPG activity, we also determined the hydrolysis degree that was calculated as the ratio of reducing sugar generation to initial pectin.

### LC-MS/MS detection of metabolites of *K. marxianus*

After 72 h of cultivation, different groups with same amount of cell thallus were collected by centrifugation at low temperature and washed three times using sterile water for next extraction of metabolites. All the extracts were pre-cooled at -20 ℃ before use. The samples were allowed to thaw at room temperature, and then transferred to a 1.5 mL tube, with the addition of 1 mL pre-cooled methanol-water (V: V = 4:1) and 4 µg/mL L-2-chlorophenylalanine as internal standard, mixing for 10 s. Subsequently, 200 µL chloroform was added and the sample mixture was allowed to evaporate, followed by ultrasonic treatment (500 W) for 3 min and ultrasonic extraction for 20 min in an ice water bath, and left overnight at -40 ℃. The resultant solution was centrifuged at 12,000 rpm at 4 ℃ for 10 min, and 800 µL supernatant was placed in a glass vial and dried in a refrigerated concentration centrifugal dryer. A 300 µL methanol-water (V: V = 1:4) mixture was added to each sample and mixed for 30 s, before leaving at -40 ℃ for 2 h. A subsequent ultrasonic extraction was performed under ice condition for 3 min, with centrifugation at 12,000 rpm for 10 min at a low temperature. Each tube containing 150 µL supernatant was collected using syringes, filtered through 0.22 μm microfilters, transferred to a LC sample vial, and stored at -80 ℃ before analysis by liquid chromatography-mass spectrometry (LC-MS).

The metabolomic analysis was performed by Shanghai Luming Biological Technology Co., Ltd (Shanghai, China). An ACQUITY UPLC I-Class plus (Waters Corp., Milford, USA) with a Q-Exactive mass spectrometer, equipped with an ACQUITY UPLC HSS T3 column (100 × 2.1 mm, 1.8 μm) and heated using an electrospray ionization (ESI) source (Thermo Fisher Scientific, Waltham, MA, USA) was employed for metabolic profiling in both ESI positive and negative ion modes. The binary gradient elution system consisted of (A) water (containing 0.1% v/v formic acid) and (B) acetonitrile (containing 0.1% v/v formic acid). Separation of metabolites was achieved using the following gradient: 0.01 min, 95% A and 5% B; 2 min, 95% A and 5% B; 4 min, 70% A and 30% B; 8 min, 50% A and 50% B; 10 min, 20% A and 80% B; 14 min, 100% B; 15 min, 100% B; 15.1 min, 95% A and 5% B; 16 min, 95% A and 5% B. The flow rate was 0.35 mL/min and the column temperature was 45 ℃. All the samples were kept at 10 ℃ and the injection volume was 5 µL. The mass analysis ranged from m/z 70 to 1050. In the full MS and HCD MS/MS scans, the resolution was set at 70,000 and 17,500, respectively, and the collision energy was set at 10, 20, and 40 eV. The following parameters were employed for mass spectrometry analysis: spray voltage = 3800 V (+) and 3200 V (−); capillary temperature = 320 °C; aux gas heater temperature = 350 °C; sheath gas flow rate = 35 arb; auxiliary gas flow rate = 8 arb; S-lens RF level = 50.

### Data pre-processing and statistical analysis

The raw LC-MS data were processed using Progenesis QI V2.3 software (Nonlinear, Dynamics, Newcastle, UK) for baseline filtration, peak identification, integration, retention time correction, peak alignment, and normalization. The compounds were identified based on the precise mass-charge ratio (m/z), secondary fragments and isotope distribution employing several databases, including The Human Metabolome Database (HMDB), Lipidmaps (V2.3), Metlin and in-house databases. The extracted data were processed to remove more than 50% of the peaks with a missing value (ion intensity = 0), and replace the zero value with half the minimum value, screening according to qualitative results for the compound. The compounds that scored less than 36 out of 60 were considered inaccurate and removed. The positive and negative ion data were combined in a data matrix, which was imported into R software for analysis. A principle component analysis (PCA) was performed to determine the overall sample distribution and analysis stability. Furthermore, the orthogonal partial least-squares-discriminant analysis (OPLS-DA) and partial least-squares-discriminant analysis (PLS-DA) were used to distinguish the metabolites in each group. In order to avoid over-fitting, a 7-fold cross-validation and 200 response permutation testing (RPT) to evaluate the quality of the model was adopted. Based on the OPLS-DA model, the applied variable importance of projection (VIP) values were used to rank the overall contribution of each variable to the group discrimination. A two-tailed Student’s T-test was also applied to determine if any differences were statistically significant. Differential metabolites with VIP > 1.0 and *p* < 0.05 were selected.

### Lipid extraction and FAs quantification

The method used in lipid extraction and determination has been described previously, and this study involved some modifications of the earlier procedure (Gao et al. 2018; Wang et al. 2022). The cell shake-flask culture (20 mL) incubated for 72 h was centrifuged at 5000 g and suspended in 2 mL 4 M hydrochloric acid with incubation at 80 °C for 2 h. Following cooling, a mixture of chloroform and methanol (V/V = 2:1) was added and vortexed for 5 min to facilitate lipid extraction. After centrifugation, the organic sub-layer was collected, and dried by a rotary evaporator (Lichen, China). The extracted compounds were transformed to fatty acid methyl esters by replenishing 2 mL hydrochloric acid-methanol solution (V/V = 1:9) at 60 °C for 1 h. The methylated compounds were obtained by adding 2 mL saturated sodium chloride and 1 mL hexane, followed by vortexing for 5 min. The resulting hexane layer was transferred to a glass vial via a 0.22 μm filter for subsequent gas chromatography (GC) analysis, using Agilent 7890 B unit equipped with a fame ionization detector (FID). An Agilent J&W DBWAXetr column (30 m×0.25 mm× 0.25 μm) was employed with nitrogen as the carrier gas. The GC analysis parameters were as follows: initial temperature = 100 °C, maintained for 0.5 min, increased to 190 °C at 5 °C/min, followed by a ramp to 220 °C at 2 °C/min and held for 10 min. The injector and detector temperatures were set at 250 and 260 °C, respectively. A 1 µL sample volume was injected in split mode at a ratio of 15:1. The FAs were identified and quantified using the commercial standards and normalized with respect to methyl nonadecanate.

## Results and discussion

### Improving pectin degradation by overexpression of *PGU1* in *K. marxianus*

*K. marxianus* has been reported as a favorable strain that has the characteristics of endoPG secretion by the cultivation in a simple medium (Siekstele et al. 1999; Jia and Wheals 2000; da Silva et al. 2005; Sieiro et al. 2009). In this study, the *K. marxianus* YKM1012 was cultivated in SD medium to evaluate endoPG secretion. As depicted in Fig. 1B, endoPG production exhibited a marked increase during the 14 h cultivation (Fig. 1B).

Next, *K. marxianus* YKM1012 was cultured at varying temperatures to assess the impact of thermal conditions on endoPG secretion. Following 14 h of cultivation, exposure to 37 ℃ markedly increased endoPG level (Fig. S1), consistent with the inherent thermotolerance of *K. marxianus* species. However, cultivation at extreme high-temperature (45 ℃) failed to yield measurable endoPG secretion (Fig. S1). This suppression may reflect an energy conservation strategy, wherein the strain prioritizes cellular maintenance over enzymatic production under suboptimal thermal conditions, as hypothesized in analogous microbial systems (Thorwall et al. 2020).

Then, to elucidate pectin degradation and utilization in *K. marxianus*, we systematically investigated YKM1012 growth dynamics under varied carbon source compositions, including 2% glucose, 0.3 glucose, 0.3% glucose + 1.7% pectin, 0.3% glucose + 1.7% D-galUA, and 2% pectin. Cultivation in glucose-limited media demonstrated reduced biomass accumulation (Fig. 1C), suggesting the carbon source dependency. Although accumulated endoPG secretion was detected in SD medium at stationary phase (Fig. 1B), supplementation with exogenous pectin failed to induce growth (Fig. 1C). These results indicated YKM1012’s inability to either hydrolyze pectin or utilize it as a carbon source.

To augment endoPG production for improved pectin utilization, we developed a genome-editing strategy targeting the *ABZ1* locus in YKM1012. In this engineered strain YKM1013, the *PGU1* gene was overexpressed by the strong TEF promoter to boost endoPG expression (Fig. 1D left). Targeted deletion of the *PGU1* gene in YKM1012 was also implemented to systematically evaluate the functional contribution of endoPG for pectin degradation (Fig. 1D right). As expected, compared to YKM1012, the engineered strain YKM1013 demonstrated a significant improvement in endoPG secretion, while the *PGU1*-knockout strain YKM1014 exhibited no detection of endoPG level (Fig. 1E). These findings confirmed that the engineered strain YKM1013 provided an effective microbial chassis for enhanced endoPG production, a critical determinant for efficient pectin degradation.

**Fig. 2:**
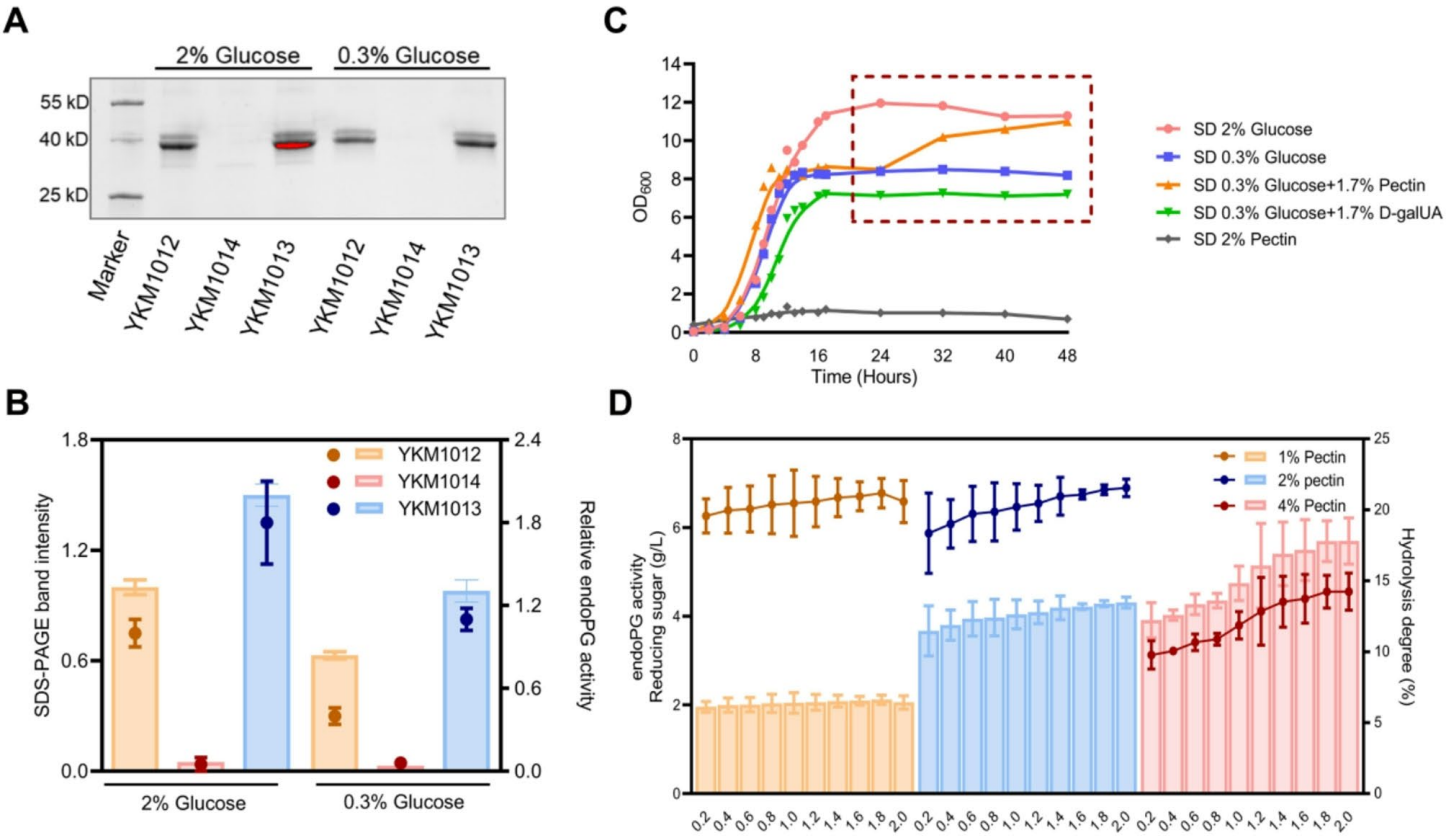
Engineering *K. marxianus* for efficient pectin hydrolysis and utilization. (**A**) The amount of endoPG secretion derived from different strains under 2% and 0.3% glucose concentrations at 30 ℃ for 20 h staining by CBB. (**B**) Determination of endoPG secretion (column, left Y-axis) and activity (symbol, right Y-axis). endoPG activity was performed by the volume ratio of 1:4 (1 mg/mL endoPG and 1% pectin) at 55 ℃ for 1 h. The sample of YKM1012 under 2% glucose was regarded as the control group. (**C**) The growth dynamics of engineered strain YKM1013 under varied carbon source compositions. The box with dashed line delineated the stationary-phase growth dynamics. (**D**) The endoPG activity (column, left Y-axis) and hydrolysis degree (symbol, right Y-axis) during pectin hydrolysis. The enzymatic assay was carried out using different combinations of YKM1013-derived endoPG (0.2-2.0 mg/mL) and pectin (1%-4%) by the volume ratio of 1:4 at 55 ℃ for 1 h. The sample without endoPG was used as the blank control, and their values were subtracted from the experimental groups for the calculation of reducing sugar

Notably, elevating glucose concentration from 0.3 to 2% in YKM1013 cultures further boosted endoPG production by 50% (Fig. 2A) (Fig. 2B, left Y-axis), with an associated twofold increase in hydrolysis capacity (Fig. 2B, right Y-axis). Furthermore, supplementation with pectin induced a distinct secondary growth response in the engineered strain YKM1013 during stationary phase (Fig. 2C), suggesting an effective utilization of pectin hydrolysate to support cell growth. Instead, taking the D-galUA components at an equal concentration, no comparable growth resurgence was a observed in YKM1013 during stationary phase (Fig. 2C). Together, these results indicated that elevated endoPG level in YKM1013 mediated efficient pectin hydrolysis, with non-D-galUA components in the hydrolysate promoting growth stimulation, while D-galUA alone showed no capacity to induce secondary growth.

A systematic investigation of pectin hydrolysis efficiency was subsequently assessed through controlled variation of pectin and YKM1013-derived endoPG concentrations. For the hydrolysis of 1% and 2% pectin, variations in endoPG concentration failed to produce any detectable effect, and the reducing sugars respectively remained at 2 g/L and 4 g/L (Fig. 2D, left Y-axis). These observations were accompanied by a constant hydrolysis degree of 20% throughout the reaction process (Fig. 2D, right Y-axis). Compared to the hydrolysis of lower pectin concentrations, the 4% pectin hydrolysis process demonstrated a marked enhancement with increasing endoPG concentrations (Fig. 2D, left Y-axis), suggesting that increased enzyme availability promotes catalytic activity through more effective enzyme-substrate engagement. However, only a 12% hydrolysis degree was achieved despite endoPG supplementation (Fig. 2D, right Y-axis), with the inhibitory effects of high viscosity in 4% pectin identified as a critical limiting factor (Xu et al. 2015). Given the multifactorial interplay in the enzyme catalytic system, the necessity for systematic optimization of reaction parameters to establish efficient pectin degradation protocols.

### Constructing the D-galUA metabolic pathway by assembling targeted heterologous enzymes in *K. marxianus*

**Fig. 3:**
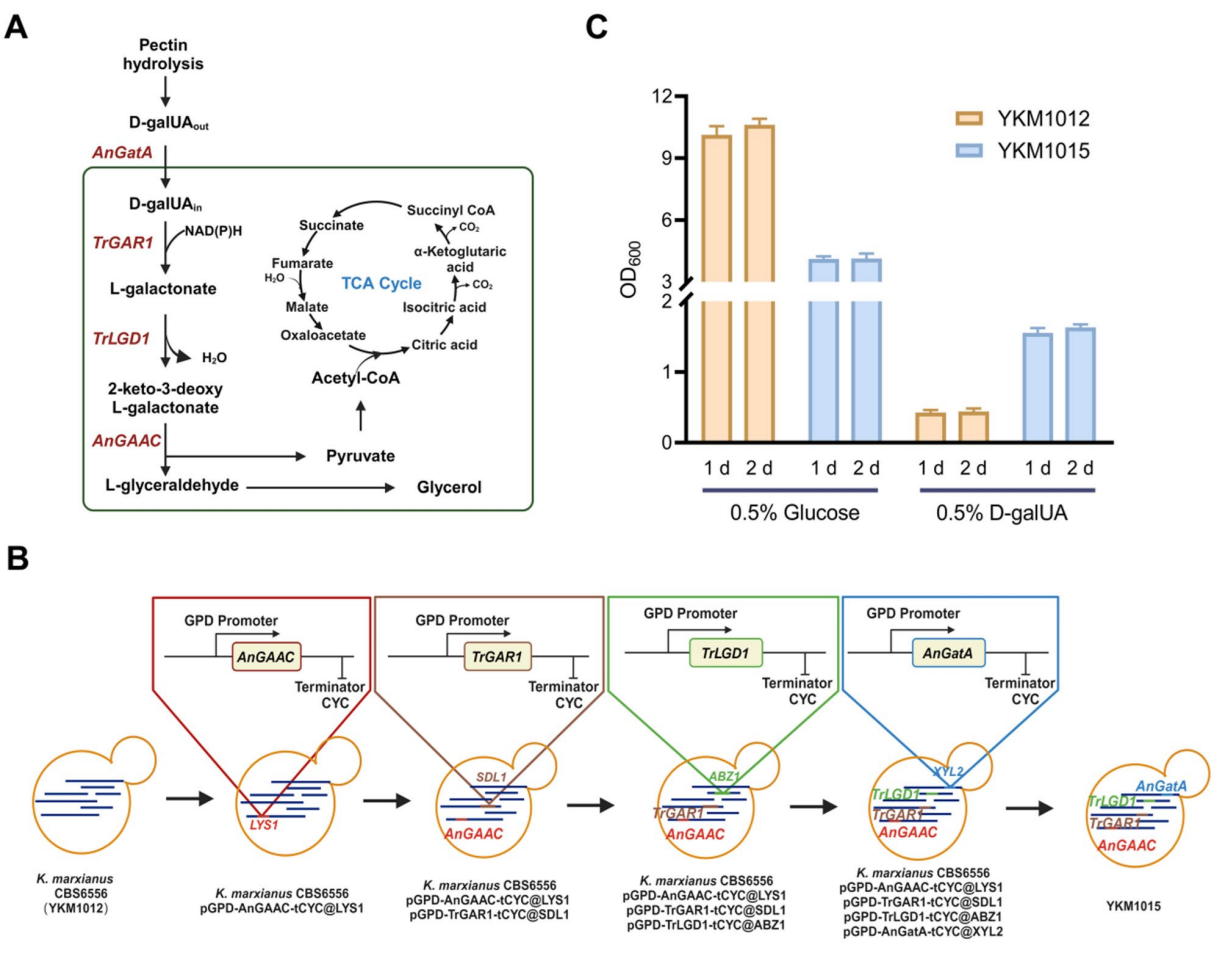
Engineering *K. marxianus* for effective utilization of D-galUA. (**A**) A simplified diagram of D-galUA metabolic pathway involving four heterologous genes derived from either *Aspergillus niger* (*An*) or *Trichoderma reesei* (*Tr*), including *AnGatA*: D-GalUA transporter; *TrGAR1*: D-galacturonate reductase; *TrLGD1*: L-galactonate dehydratase; *AnGAAC*: 2-keto-3-deoxy-L-galactonate aldolase. (**B**) Construction process of engineered strain by successive integration of four heterologous genes based on the reference strain YKM1012 through the gene sites of *LYS1*, *SDL1*, *ABZ1*, and *XYL2*. (**C**) The cell growth dynamics of YKM1012 and YKM1015 by the separate cultivation in glucose and D-galUA

While the engineered strain YKM1013 demonstrated selective hydrolysis capability to mobilize a small amount of available sugars from pectin hydrolysate, most of the D-galUA components remained unutilized (Fig. 2C), indicating fundamental constraints in its metabolic pathways. In previous reports, the heterologous genes involved in the D-galUA metabolic pathway were shown to be active in *S. cerevisiae*, including *GAR1*, *LGD1*, *GAAC*, and *GatA* (Fig. 3A) (Biz et al. 2016; Protzko et al. 2018; Perpelea et al. 2022). We then engineered *K. marxianus* to drive D-galUA metabolic flux integrating *TrGAR1*, *TrLGD1*, *AnGAAC*, and *AnGatA* into the high-efficiency loci *SDL1*, *ABZ1*, *LYS1*, and *XYL2*, respectively (Fig. 3B) (Li et al. 2021), generating the engineered strain YKM1015 (Fig. 3B). Finally, the contribution of D-galUA was identified in YKM1015 by separately feeding 0.5% glucose and D-galUA. Notably, an excellent performance of cell growth for YKM1015 in D-galUA cultivation demonstrated a 3.6-fold difference comparing with YKM1012, which preferred to grow in glucose cultivation (Fig. 3C). Hence, the constructed strain exhibited a significant potential to successfully channel metabolic flux into the D-galUA pathway, facilitating an effective utilization of D-galUA components.

### Optimizing pectin degradation efficiency by application of engineered *K. marxianus*

To systematically explore the optimal hydrolysis conditions, a rational strategy was implemented, including strain culture parameter, thermal stability analysis, and operational reaction system. Firstly, considering the elevated endoPG secretion under 37 ℃ cultivation (Fig. S1), the culture parameter for YKM1013 was assessed through temperature-dependent analysis of endoPG hydrolytic activity. Nevertheless, extended cultivation (> 10 h) at 37 °C resulted in markedly reduced activity of endoPG compared to that observed at 30 °C (Fig. S2). Further discovery exhibited that prolonged incubation (24 h) at 30 ℃ unexpectedly induced activity and followed by progressive deactivation (Fig. S2). Consequently, a 24 h cultivation regimen at 30 ℃ was established as the optimal protocol for achieving sustained endoPG ability.

**Fig. 4:**
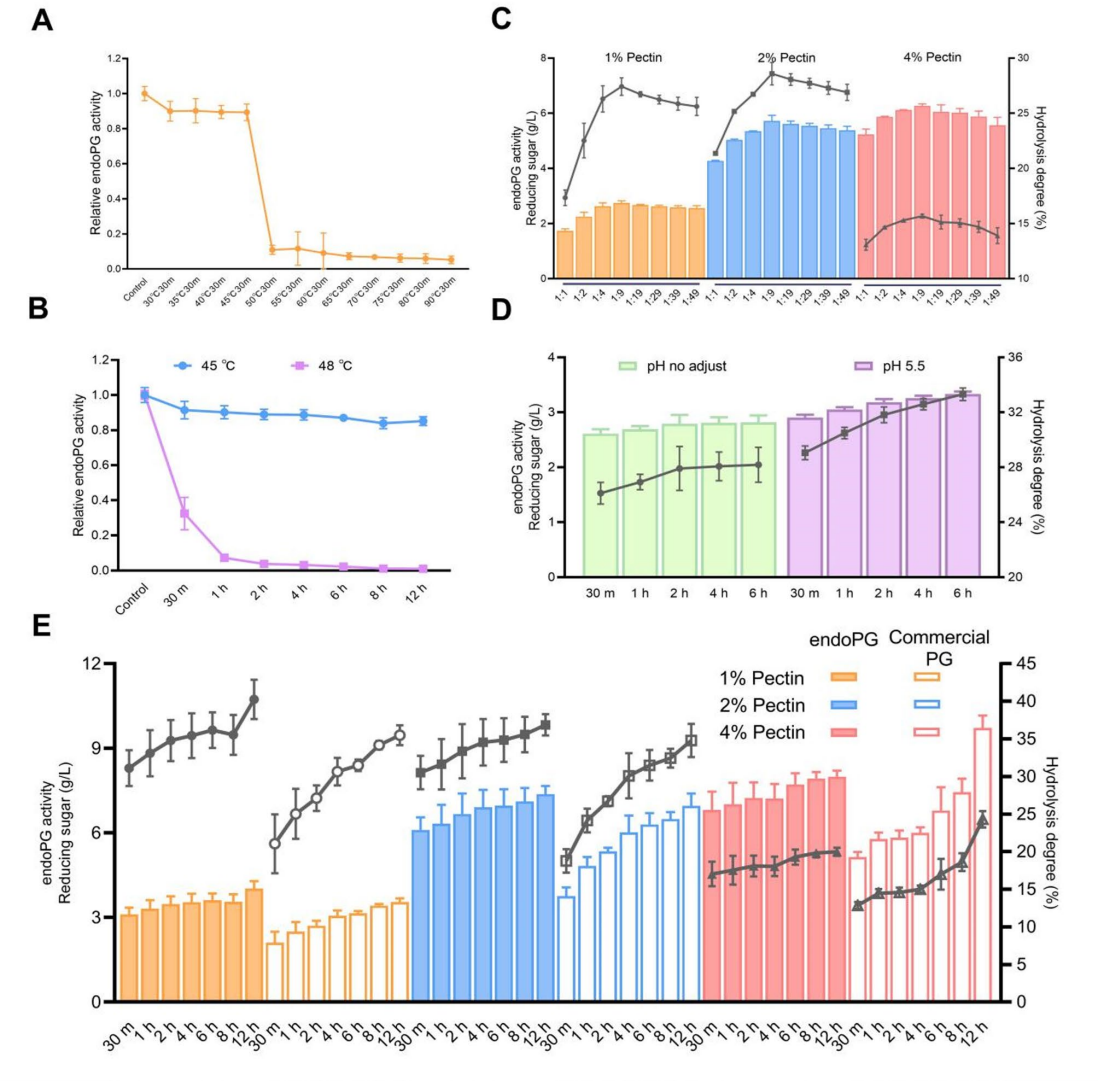
Optimization conditions for improving pectin hydrolysis efficiency. (**A**) Evaluation of thermal stability of endoPG derived from YKM1013 by the volume ratio of 1:4 (0.2 mg/mL endoPG and 1% pectin) at 55 ℃ for 30 min by pre-treatment at varied temperatures. The sample without pre-treatment was used as the blank control. (**B**) Comparison of thermal stability of endoPG derived from YKM1013 by the volume ratio of 1:4 (0.2 mg/mL endoPG and 1% pectin) at 45 ℃ for 30 min through time-course pre-treatment between 45 ℃ and 48 ℃. The samples without pre-treatment were used as the blank controls. (**C**) The concentration of reducing sugar (column, left Y-axis) and hydrolysis degree (symbol, right Y-axis) among varied ratios of endoPG (0.6 mg/mL) and pectin at 45 ℃ for 30 min. (**D**) The endoPG activity in different pH environments by the volume ratio of 1:9 (0.6 mg/mL endoPG and 1% pectin) at 45 ℃ for varied durations. The reaction environment without pH modification and pH-modified 5.5. (**E**) Comparison of endoPG and commercial PG in varied concentrations of endoPG and pectin. The enzymatic assay was incubated by the volume ratio of 1:9 (endoPG and pectin) at 45 ℃ for varied durations with pH value as 5.5. The two types of enzymes with the same concentration (0.6 mg/mL) were applied for hydrolysis reaction. Left Y-axis, Concentration of reducing sugar; Right Y-axis, Hydrolysis degree

Subsequently, to guide the compromise temperature balancing enzymatic efficiency and stability, a systematic characterization of endoPG thermostability was conducted. A marked response was identified a narrow activity window between 45 and 50 ℃ range over a 30 min pre-treatment period (Fig. 4A). Further measurement indicated that the system was thermally stable up to 12 h at 45 ℃, with evidence of a loss of activity at 48 ℃ within 30 min (Fig. 4B). While prior investigations established 35–40 ℃ as the optimal thermal range for incubation (da Silva et al. 2005; Masoud and Jespersen 2006), our systematic thermal profiling revealed unexpected enzymatic behaviors. Considering the differences in incubation temperatures, we then investigated pectin hydrolysis activity by measuring reducing sugar levels. Notably, the reducing sugar concentration exhibited no significant variation across the temperature range of 30–45 ℃ (Fig. S3). Remarkably, 70% of the endoPG activity persisted following 48 h thermal challenge at 45 ℃, even half residual activity remaining after 120 h exposure (Fig. S4). Consequently, these findings motivated the implementation of 45 ℃ as the operational temperature for the pectin hydrolysis system, strategically balancing catalytic efficiency with long-term system durability.

Finally, to further improve pectin hydrolysis efficiency, a comprehensive multi-parameter optimization was implemented, incorporating both stoichiometric optimization of pectin-endoPG ration and ph-mediated modulation. Maintaining a total reaction volume of 100 µL, systematic variation of the pectin-to-endoPG ratio demonstrated an inverse proportionality across an endoPG concentration range of 10–50 µL (Fig. 4C). Further reduction in endoPG concentration resulted in diminished activity of pectin hydrolysis (Fig. 4C). The highest hydrolysis degree (28%) was attained at a 1:9 (v/v) endoPG-to-pectin ratio in a 2% pectin hydrolysis system (Fig. 4C, right Y-axis). Previous works have pointed out that the endoPG secreted by these yeasts exhibited activity within a pH range of 4.0 to 6.0 (Moyo et al. 2003; da Silva et al. 2005). In the case of *K. marxianus* strain 166 and CCT 3172, the highest activity of endoPG secretion was recorded at pH 5.5 (da Silva et al. 2005; Masoud and Jespersen 2006). This study evaluated two distinct reaction environments, both adjusted to pH 5.5 using NaOH. For the one type, endoPG was dissolved in PBS while 1% pectin was prepared in distilled water, yielding a 33% hydrolysis degree in the 1% pectin hydrolysis assay (Fig. 4D, right Y-axis). For the other type, both endoPG and 1% pectin were dissolved in sodium acetate buffer, however, it showed significantly suppressed enzymatic activity, achieving only ~ 20% conversion (Fig. S5). Together, these results demonstrated that at the optimal pH of 5.5, endoPG exhibited higher hydrolysis efficiency in PBS than in sodium acetate buffer.

In summary, employing a systematic optimization strategy, we comprehensively evaluated the influence of key reaction parameters, including temperature, enzyme-substrate ratio, and pH, on the hydrolysis efficiency of YKM1013-derived endoPG relative to its commercial counterpart (Fig. 4E). The endoPG demonstrated significantly greater catalytic consistency across incubation durations and markedly enhanced activity relative to the commercial PG in short-term reactions (Fig. 4E). Notably, under optimized reaction conditions (45 °C, 2 h) for 2% pectin hydrolysis, the endoPG demonstrated substantial improvements, yielding 75% and 65% increases in reducing sugar concentration and pectin hydrolysis degree, respectively (Fig. 2D) (Fig. 4E). These findings indicated that the multi-parameter systematic optimization substantially improved enzymatic efficiency, with YKM1013-derived endoPG enabling a particular potential for catalytic applications.

### Developing a modular system for pectin bioconversion by two engineered strains

**Fig. 5:**
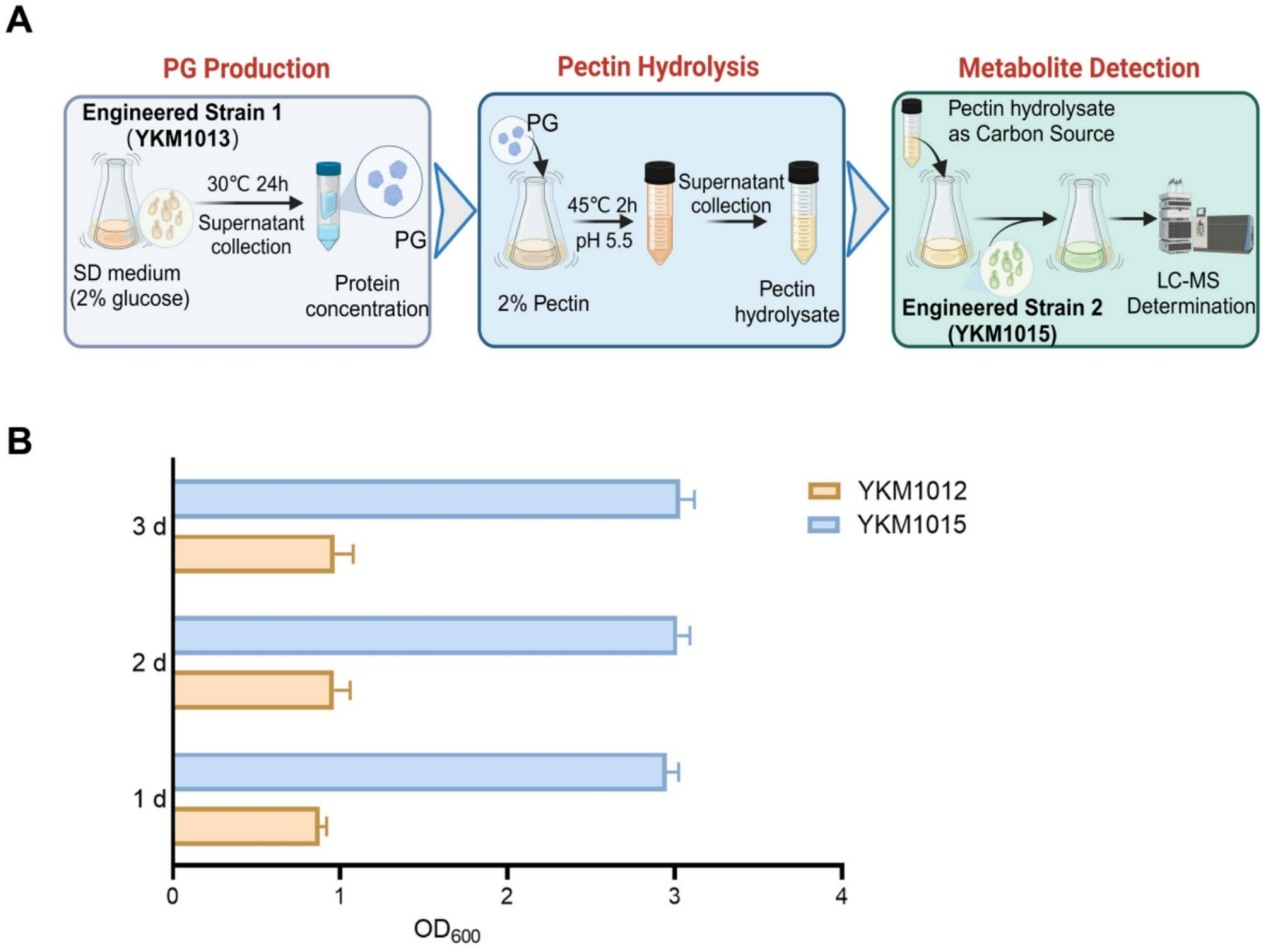
Modular utilization of two engineered *K. marxianus* strains for pectin bioconversion. (**A**) The established process of pectin bioconversion through modular utilization of two engineered *K. marxianus* strains. Engineered strain 1: YKM1013; Engineered strain 2: YKM1015. (**B**) The cell growth of YKM1012 and YKM1015 by the cultivation in pectin hydrolysate

Having optimized the pectin hydrolysis system, we next set out to develop pectin bioconversion processing by modular utilization of two engineered strains. Firstly, the endoPG production commenced with cultivation of engineered strain 1 (YKM1013) in SD medium at 30 °C for 24 h. After incubation, the harboring cells were removed via centrifugation at 4 °C, followed by supernatant concentration using 10 kDa ultrafiltration membranes. The crude enzyme preparation underwent buffer exchange to phosphate buffer solution (PBS), achieving a final endoPG protein concentration of 0.6 mg/mL (Fig. 5A). Then, 2% pectin was hydrolyzed by employing 0.6 mg/mL endoPG at 45 °C for 2 h, and the pectin hydrolysate was clarified via high-speed centrifugation at 4 °C (Fig. 5A). Lastly, clarified hydrolysate was supplemented with 5× stored Yeast Nitrogen Base without Amino Acids and Yeast Amino Acids Supplement to incubate engineered strain 2 (YKM1015) at 30 °C for 72 h by initial OD 0.05 for metabolites determination (Fig. 5A). The strain YKM1012 was served as the control group for metabolic profiling analysis by the cultivation in pectin hydrolysate as the carbon source. As a result, the harboring YKM1015 demonstrated a twofold increase biomass relative to YKM1012 (Fig. 5B), directly correlating with the contribution of D-GalUA utilization. Thus, these results indicated that this proposed dual-strain modular strategy can facilitate pectin bioconversion via synergistic enzymatic coordination.

### Establishing strategy enhanced pectin-based lipid metabolism and FAs production

**Fig. 6:**
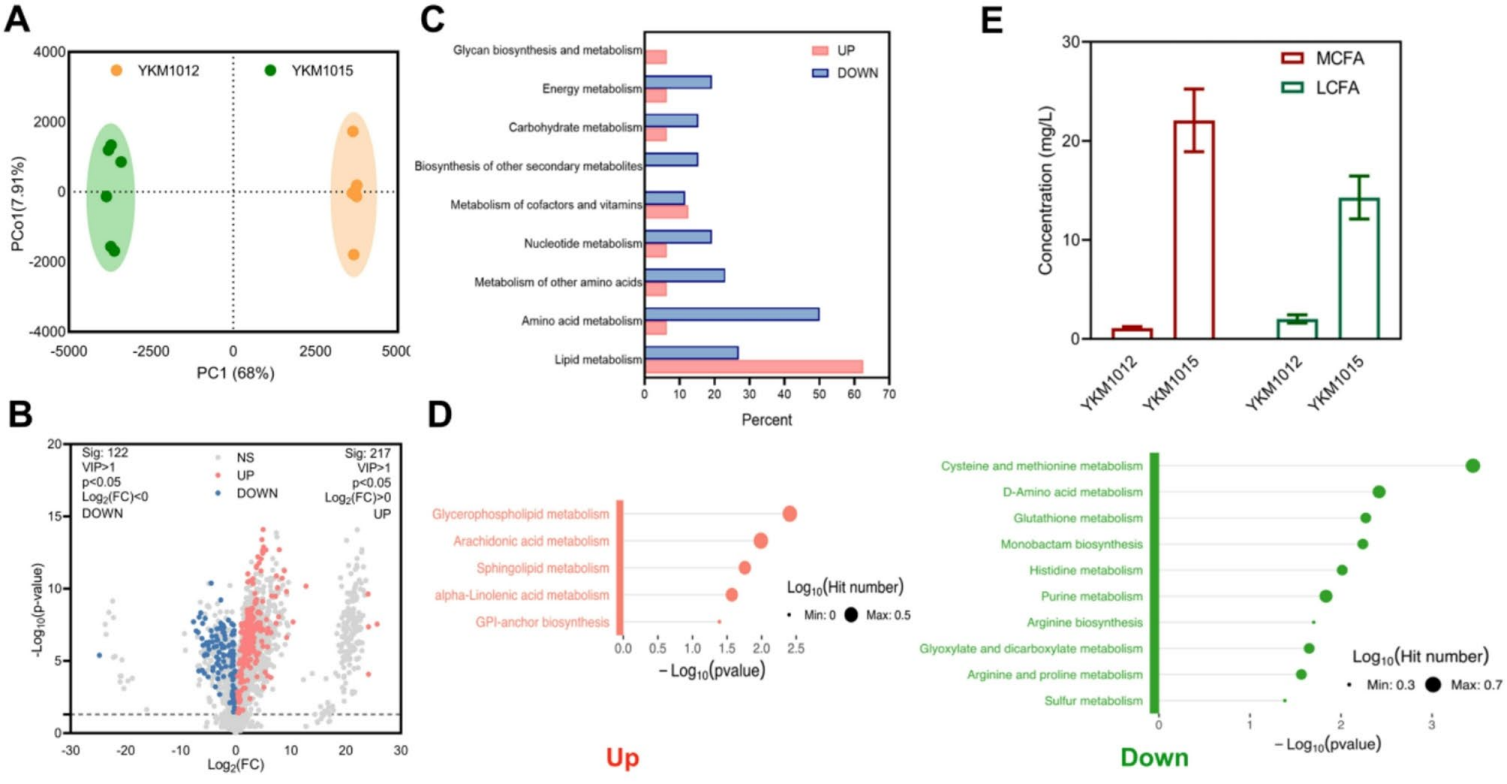
Transformation of lipid metabolism flux in pectin bioconversion for FAs production. (**A**) OPLS-DA load diagram and (**B**) Volcano plot of metabolic profiles between YKM1012 and YKM1015. (**C**) Summary of classified metabolism pathway of significantly up- and down-regulated metabolites between YKM1012 and YKM1015. (**D**) Detail of metabolism pathway enrichment of up-regulated (*left*) and down-regulated (*right*) metabolites (VIP > 1, *p* < 0.05) between YKM1012 and YKM1015. (**E**) The concentrations of MCFA and LCFA between YKM1012 and YKM1015

To assess metabolic reprogramming performance of in pectin bioconversion, we analyzed the metabolic profiles of different strains. Here, a total of over 3,000 compounds were detected (Supplementary Data 1) and the chromatograms were presented in Fig. S6. Incorporating considerations of cellular growth dynamics and metabolic regulation, multivariate analysis revealed a marked divergence in metabolic profiles between YKM1012 and YKM1015 (Fig. 6A). Specifically, compared with YKM1012, YKM1015 exhibited significant up-regulation of 217 metabolites concurrent with suppression of 122 metabolites (Fig. 6B). Unexpectedly, pathway enrichment analysis revealed significant accumulation patterns in lipid metabolism (Fig. 6C), indicating that the genetic modifications in YKM1015 redirected metabolic flux toward lipid biosynthesis. However, metabolic reprogramming in YKM1015 resulted in down-regulation of amino acids metabolism pathways (Fig. 6C). Analysis of significantly enriched pathways revealed pronounced metabolite accumulation in specific lipid biosynthesis pathways, particularly involving glycerophospholipid metabolism, arachidonic acid metabolism, α-linolenic acidmetabolism, and sphingolipid metabolism (Fig. 6D left). Conversely, the majority of down-regulated metabolites were associated with amino acid biosynthesis pathways (Fig. 6D right).

Referring to the previous studies, *T. reesei* LGD1 fused with yellow fluorescent protein (Venus-LGD1, V-TrLGD1) was shown to improve a 60-fold enzyme activity in vitro (Biz et al. 2016; Protzko et al. 2018). Comparative analysis revealed that the V-TrLGD1-expressing strain YKM1016 exhibited an analogous manner to YKM1015 with respect to cell growth despite distinct genetic modifications (Fig. 5B, S7A). Correspondingly, the metabolic profiles revealed a striking phenotypic divergence between YKM1012 and YKM1016 (Fig. S7B). Notably, the YKM1016 and YKM1015 exhibited the metabolic convergence characterized by increased lipid metabolism and repressed amino acid metabolism (Fig. 6C, S7C, and S7D). Hence, this metabolic reprogramming pattern suggested host-specific flux partitioning mechanisms in *K. marxianus* that overrode heterologous LGD level. Ultimately, the heterologous metabolic pathway for pectin bioconversion was successfully designed in *K. marxianus*, yielding 22 mg/L mid-chain fatty acids (MCFA) and 14 mg/L long-chain fatty acids (LCFA), pointing at an increasing of 19-fold and 6-fold, respectively (Fig. 6E). Collectively, this pectin utilization route can be tailored to generate a range of products and serve as an effective platform for pectin-rich biomass bioconversion.

In contrast to *Yarrowia lipolytica*, a well-studied cell factory for producing lipids via metabolic engineering strategy (Lazar et al. 2018; Wang et al. 2020; Chen et al. 2021), little research referring to lipids has been carried out in *K. marxianus* so far. The phosphatidylcholines (PCs), common in the membranes of most eukaryotes, are involved in the sphingolipid and glycerophospholipid metabolisms to determine the physical properties and strictly regulated (de Kroon 2007). Thereby, alteration of PCs implied the difference of membrane profile when utilizing pectin hydrolysate. Although most of the information referring to unsaturated fatty acid (UFA) metabolism has been obtained from mammalian biology, the relevant compounds can also be produced by yeasts, such as *Candida albicans*, *Cryptococcus neoformans* (Deva et al. 2000; Erb-Downward and Huffnagle 2007), and *Y. lipolytica* (Liu et al. 2019). Little information is, however, available regarding the metabolites involved in α-linolenic acid and arachidonic acid production in *K. marxianus*. In yeast, the availability of amino acids is important in cell growth and regulatory processes in a complicated network (Broach 2012; Takagi 2019). Therefore, the engineered strain YKM1015 was possibly influenced by an inhibited cell growth and regulatory processes.

Comparative metabolomic profiling revealed substrate-dependent flux bifurcations between pectin hydrolysate and glucose cultivation (Fig. 6C, Fig. S8). The glucose-based metabolism triggered extensive rerouting of central carbon flow, with pronounced differential regulation in amino acid flux, including the enhanced histidine metabolism and lysine degradation, and repressed valine, leucine and isoleucine biosynthesis (Fig. S8). This substrate-directed metabolic channeling aligns with reported carbon source modulation of microbial growth kinetics (Broach 2012). Taken together, these adaptive metabolic features position *K. marxianus* as a modular chassis for valorizing pectin-rich feedstocks into high-value products through substrate-regulated pathway orchestration.

## Conclusions

In summary, this study develops a modular processing system through rational application of two engineered *K. marxianus* strains for synergistic pectin valorization, resulting in a 65% improvement of hydrolysis degree and enhancing lipid metabolism flux with an increasing of 19-fold in MCFA and 6-fold in LCFA production. The strategy creates a feasible carbon-efficient bioconversion route of pectin-rich materials into high-value chemicals, advancing the valorization of underutilized pectin-rich biomass by diverse microbial cell factories in feedstock resources utilization.

## Supplementary Information

Below is the link to the electronic supplementary material.

Supplementary Material 1

## Data Availability

The data and materials that support the findings of this study are available from the corresponding author upon reasonable request.

## Declarations

### Associated content

Supporting information. endoPG secretion derived from YKM1012 (Fig. S1); the activity of endoPG derived from YKM1013 (Fig. S2, S3, S5); thermal stability of endoPG derived from YKM1013 (Fig. S4); chromatograms for LC-MS/MS detection of YKM1012 and YKM1015 (Fig. S6); metabolic profile between YKM1012 and YKM1016 strains (Fig. S7); differential metabolites between YKM1012 and YKM1015 strains by the cultivation in 2% glucose (Fig. S8).

## Abbreviations

5’-FOA: 5-fluoroorotic acid
*A. niger*: *Aspergillus niger*
CBB: Coomassie brilliant blue
D-galUA: D-galacturonic acid
DNS: 3,5-dinitrosalicylic acid
DO: Dropout
*E. coli*: *Escherichia coli*
endoPG: Endo-polygalacturonase
ESI: Electrospray ionization
FID: Fame ionization detector
GAAC: 2-keto-3-deoxy-L-galactonate aldolase
GAR1: D-galacturonate reductase
GatA: D-GalUA transporter
GC: Gas chromatography
GRAS: Generally recognized as safe
HMDB: The Human Metabolome Database
*K. marxianus*: *Kluyveromyces marxianus*
LB: Luria–Bertani
LCFA: Long-chain fatty acids
LC-MS: Liquid chromatography-mass spectrometry
LGD1: L-galactonate dehydratase
MCFA: Mid-chain fatty acids
OD: Optical density
OPLS-DA: Orthogonal partial least-squares-discriminant analysis
PBS: Phosphate buffer solution
PCA: Principle component analysis
PCR: Polymerase chain reaction
PCs: Phosphatidylcholines
PL: Pectin lyase
PLS-DA: Partial least-squares-discriminant analysis
PME: Pectin methylesterase
RPT: Response permutation testing
*S. cerevisiae*: *Saccharomyces cerevisiae*
SD: Synthetic defined
UFA: Unsaturated fatty acids
VIP: Variable importance of projection
V-TrLGD1: LGD1 fused with yellow fluorescent protein
YPD: Yeast-extract peptone dextrose

## References

[CR1] Bilal M, Ji L, Xu Y, Xu S, Lin Y, Iqbal HMN, Cheng H (2022) Bioprospecting *Kluyveromyces Marxianus* as a robust host for industrial biotechnology. Front Bioeng Biotechnol 10:851768 35519613 10.3389/fbioe.2022.851768 PMC9065261

[CR2] Biz A, Sugai-Guérios MH, Kuivanen J, Maaheimo H, Krieger N, Mitchell DA, Richard P (2016) The introduction of the fungal D-galacturonate pathway enables the consumption of D-galacturonic acid by *Saccharomyces cerevisiae*. Microb Cell Fact 15(1):144 27538689 10.1186/s12934-016-0544-1 PMC4990863

[CR3] Bonnin E, Garnier C, Ralet MC (2014) Pectin-modifying enzymes and pectin-derived materials: applications and impacts. Appl Microbiol Biotechnol 98(2):519–532 24270894 10.1007/s00253-013-5388-6

[CR4] Broach JR (2012) Nutritional control of growth and development in yeast. Genetics 192(1):73–105 22964838 10.1534/genetics.111.135731 PMC3430547

[CR5] Chavan P, Singh AK, Kaur G (2019) Recent progress in the utilization of industrial waste and by-products of citrus fruits: A review. J Food Process Eng

[CR6] Chen L, Yan W, Qian X, Chen M, Zhang X, Xin F, Zhang W, Jiang M, Ochsenreither K (2021) Increased lipid production in *Yarrowia lipolytica* from acetate through metabolic engineering and cosubstrate fermentation. ACS Synth Biol 10(11):3129–3138 34714052 10.1021/acssynbio.1c00405

[CR7] da Silva EG, de Fátima Borges M, Medina C, Piccoli RH, Schwan RF (2005) Pectinolytic enzymes secreted by yeasts from tropical fruits. FEMS Yeast Res 5(9):859–865 15925314 10.1016/j.femsyr.2005.02.006

[CR9] de Kroon AI (2007) Metabolism of phosphatidylcholine and its implications for lipid acyl chain composition in *Saccharomyces cerevisiae*. Biochim Biophys Acta 1771(3):343–352 17010666 10.1016/j.bbalip.2006.07.010

[CR8] Dekker WJC, Ortiz-Merino RA, Kaljouw A, Battjes J, Wiering FW, Mooiman C, Torre P, Pronk JT (2021) Engineering the thermotolerant industrial yeast *Kluyveromyces Marxianus* for anaerobic growth. Metab Eng 67:347–364 34303845 10.1016/j.ymben.2021.07.006

[CR10] Deva R, Ciccoli R, Schewe T, Kock JL, Nigam S (2000) Arachidonic acid stimulates cell growth and forms a novel oxygenated metabolite in *Candida albicans*. Biochim Biophys Acta 1486(2–3):299–311 10903481 10.1016/s1388-1981(00)00073-1

[CR11] Erb-Downward JR, Huffnagle GB (2007) *Cryptococcus neoformans* produces authentic prostaglandin E_2_ without a cyclooxygenase. Eukaryot Cell 6(2):346–350 17158733 10.1128/EC.00336-06 PMC1797952

[CR12] Erian AM, Sauer M (2022) Utilizing yeasts for the conversion of renewable feedstocks to sugar alcohols - a review. Bioresour Technol 346:126296 34798255 10.1016/j.biortech.2021.126296

[CR14] Gao Q, Cao X, Huang YY, Yang JL, Chen J, Wei LJ, Hua Q (2018) Overproduction of fatty acid Ethyl esters by the oleaginous yeast *Yarrowia lipolytica* through metabolic engineering and process optimization. ACS Synth Biol 7(5):1371–1380 29694786 10.1021/acssynbio.7b00453

[CR13] Gao J, Yu W, Li Y, Jin M, Yao L, Zhou YJ (2023) Engineering co-utilization of glucose and xylose for chemical overproduction from lignocellulose. Nat Chem Biol 19(12):1524–1531 37620399 10.1038/s41589-023-01402-6

[CR15] Grohmann K, Baldwin EA, Buslig BS (1994) Production of ethanol from enzymatically hydrolyzed orange Peel by the yeast *Saccharomyces cerevisiae*. Appl Biochem Biotechnol 45–46:315–327 8010764 10.1007/BF02941808

[CR16] Huisjes EH, Luttik MA, Almering MJ, Bisschops MM, Dang DH, Kleerebezem M, Siezen R, van Maris AJ, Pronk JT (2012) Toward pectin fermentation by *Saccharomyces cerevisiae*: expression of the first two steps of a bacterial pathway for D-galacturonate metabolism. J Biotechnol 162(2–3):303–310 23079077 10.1016/j.jbiotec.2012.10.003

[CR17] Jia J, Wheals A (2000) Endopolygalacturonase genes and enzymes from *Saccharomyces cerevisiae* and *Kluyveromyces Marxianus*. Curr Genet 38(5):264–270 11191210 10.1007/s002940000160

[CR18] Lazar Z, Liu N, Stephanopoulos G (2018) Holistic approaches in lipid production by *Yarrowia lipolytica*. Trends Biotechnol 36(11):1157–1170 30006239 10.1016/j.tibtech.2018.06.007

[CR20] Li M, Lang XY, Moran Cabrera M, De Keyser S, Sun X, Da Silva N, Wheeldon I (2021) CRISPR-mediated multigene integration enables Shikimate pathway refactoring for enhanced 2-phenylethanol biosynthesis in *Kluyveromyces Marxianus*. Biotechnol Biofuels 14(1):3 33407831 10.1186/s13068-020-01852-3 PMC7788952

[CR21] Li S, Su C, Fang M, Cai D, Deng L, Wang F, Liu J (2023) Overproduction of palmitoleic acid from corn Stover hydrolysate by engineered *Saccharomyces cerevisiae*. Bioresour Technol 382:129211 37217143 10.1016/j.biortech.2023.129211

[CR19] Li J, Peng C, Mao A, Zhong M, Hu Z (2024) An overview of microbial enzymatic approaches for pectin degradation. Int J Biol Macromol 254:127804 37913880 10.1016/j.ijbiomac.2023.127804

[CR22] Liang C, Gui X, Zhou C, Xue Y, Ma Y, Tang SY (2015) Improving the thermoactivity and thermostability of pectatelyase from *Bacillus pumilus* for Ramie degumming. Appl Microbiol Biotechnol 99(6):2673–2682 25287558 10.1007/s00253-014-6091-y

[CR23] Liu HH, Wang C, Lu XY, Huang H, Tian Y, Ji XJ (2019) Improved production of arachidonic acid by combined pathway engineering and synthetic enzyme fusion in *Yarrowia lipolytica*. J Agric Food Chem 67(35):9851–9857 31418561 10.1021/acs.jafc.9b03727

[CR24] Löbs AK, Lin JL, Cook M, Wheeldon I (2016) High throughput, colorimetric screening of microbial ester biosynthesis reveals high Ethyl acetate production from *Kluyveromyces Marxianus* on C5, C6, and C12 carbon sources. Biotechnol J 11(10):1274–1281 27528369 10.1002/biot.201600060

[CR25] Ma X, Wang DL, Chen WJ, Ismail BB, Wang WJ, Lv RL, Ding T, Ye XQ, Liu DH (2018) Effects of ultrasound pretreatment on the enzymolysis of pectin: kinetic study, structural characteristics and anti-cancer activity of the hydrolysates. Food Hydrocolloids 79:90–99

[CR26] Masoud W, Jespersen L (2006) Pectin degrading enzymes in yeasts involved in fermentation of *Coffea Arabica* in East Africa. Int J Food Microbiol 110(3):291–296 16784790 10.1016/j.ijfoodmicro.2006.04.030

[CR27] Miller G (1959) Use of dinitrosalicyclic acid reagent for determination of reducing sugars. Anal Chem 31:426–428

[CR28] Mohnen D (2008) Pectin structure and biosynthesis. Curr Opin Plant Biol 11(3):266–277 18486536 10.1016/j.pbi.2008.03.006

[CR29] Motter FA, Kuivanen J, Keränen H, Hilditch S, Penttilä M, Richard P (2014) Categorisation of sugar acid dehydratases in *Aspergillus Niger*. Fungal Genet Biol 64:67–72 24382357 10.1016/j.fgb.2013.12.006

[CR30] Moyo S, Gashe BA, Collison EK, Mpuchane S (2003) Optimising growth conditions for the pectinolytic activity of *Kluyveromyces wickerhamii* by using response surface methodology. Int J Food Microbiol 85(1–2):87–100 12810274 10.1016/s0168-1605(02)00503-2

[CR31] Perpelea A, Wijaya AW, Martins LC, Rippert D, Klein M, Angelov A, Peltonen K, Teleki A, Liebl W, Richard P, Thevelein JM, Takors R, Sá-Correia I, Nevoigt E (2022) Towards valorization of pectin-rich agro-industrial residues: engineering of *Saccharomyces cerevisiae* for co-fermentation of d-galacturonic acid and glycerol. Metab Eng 69:1–14 34648971 10.1016/j.ymben.2021.10.001

[CR32] Protzko RJ, Latimer LN, Martinho Z, de Reus E, Seibert T, Benz JP, Dueber JE (2018) Engineering *Saccharomyces cerevisiae* for co-utilization of D-galacturonic acid and D-glucose from citrus Peel waste. Nat Commun 9(1):5059 30498222 10.1038/s41467-018-07589-w PMC6265301

[CR33] Sharma P, Vishvakarma R, Gautam K, Vimal A, Kumar Gaur V, Farooqui A, Varjani S, Younis K (2022) Valorization of citrus Peel waste for the sustainable production of value-added products. Bioresour Technol 351:127064 35351555 10.1016/j.biortech.2022.127064

[CR34] Sieiro C, Sestelo AB, Villa TG (2009) Cloning, characterization, and functional analysis of the EPG1-2 gene: a new allele coding for an endopolygalacturonase in *Kluyveromyces Marxianus*. J Agric Food Chem 57(19):8921–8926 19725536 10.1021/jf900352q

[CR35] Siekstele R, Bartkeviciute D, Sasnauskas K (1999) Cloning, targeted disruption and heterologous expression of the *Kluyveromyces Marxianus* endopolygalacturonase gene (EPG1). Yeast 15(4):311–322 10206190 10.1002/(sici)1097-0061(19990315)15:4<311::aid-yea379>3.0.co;2-9

[CR36] Takagi H (2019) Metabolic regulatory mechanisms and physiological roles of functional amino acids and their applications in yeast. Biosci Biotechnol Biochem 83(8):1449–1462 30712454 10.1080/09168451.2019.1576500

[CR37] Thorwall S, Schwartz C, Chartron JW, Wheeldon I (2020) Stress-tolerant non-conventional microbes enable next-generation chemical biosynthesis. Nat Chem Biol 16(2):113–121 31974527 10.1038/s41589-019-0452-x

[CR38] Wagner JM, Alper HS (2016) Synthetic biology and molecular genetics in non-conventional yeasts: current tools and future advances. Fungal Genet Biol 89:126–136 26701310 10.1016/j.fgb.2015.12.001

[CR40] Wang J, Ledesma-Amaro R, Wei Y, Ji B, Ji XJ (2020) Metabolic engineering for increased lipid accumulation in *Yarrowia lipolytica* - A review. Bioresour Technol 313:123707 32595069 10.1016/j.biortech.2020.123707

[CR39] Wang K, Shi TQ, Wang J, Wei P, Ledesma-Amaro R, Ji XJ (2022) Engineering the lipid and fatty acid metabolism in *Yarrowia lipolytica* for sustainable production of high oleic oils. ACS Synth Biol 11(4):1542–1554 35311250 10.1021/acssynbio.1c00613

[CR41] Wikandari R, Hasniah N, Taherzadeh MJ (2022) The role of filamentous fungi in advancing the development of a sustainable circular bioeconomy. Bioresour Technol 345:126531 34896535 10.1016/j.biortech.2021.126531

[CR42] Williams DL, Schückel J, Vivier MA (2019) Grape pomace fermentation and cell wall degradation by *Kluyveromyces Marxianus* Y885. Bio Eng J 150:107282

[CR43] Wu YT, Pereira M, Venâncio A, Teixeira J (2000) Recovery of endo-polygalacturonase using polyethylene glycol-salt aqueous two-phase extraction with polymer recycling. Bioseparation 9(4):247–254 11321523 10.1023/a:1008171105319

[CR44] Xiang T, Yang R, Li L, Lin H, Kai G (2024) Research progress and application of pectin: A review. J Food Sci 89(11):6985–7007 39394044 10.1111/1750-3841.17438

[CR45] Xu S, Qin X, Liu B, Zhang DQ, Zhang YH (2015) An acidic pectin lyase from *Aspergillus Niger* with favourable efficiency in fruit juice clarification. Lett Appl Microbiol 60(2):181–187 25382689 10.1111/lam.12357

[CR46] Yuan YC, Yu BF, Zhou XZ, Qiao H, Lian JZ, Lang XY, Yao Y (2024) Engineering living material for controlled fragrance release utilizing *Kluyveromyces Marxianus* CBS6556 and adaptive hydrogel. ACS Synth Biol 13(10):3188–3196 39099325 10.1021/acssynbio.4c00229

[CR47] Zhang K, Jiang Z, Li X, Wang D, Hong J (2024) Enhancing simultaneous saccharification and co-fermentation of corncob by *Kluyveromyces Marxianus* through overexpression of putative transcription regulator. Bioresour Technol 399:130627 38522677 10.1016/j.biortech.2024.130627

