# Supplementary material for: Engineering a modular pectin-to-lipids bioconversion system using two Kluyveromyces marxianus strains
Source: Bioresour Bioprocess. 2025 Aug 12;12(1):87. doi: 10.1186/s40643-025-00927-z (PMC12343453; doi:10.1186/s40643-025-00927-z)
Supplement: Supplementary file 1 — Supplementary Material 1 [file 40643_2025_927_MOESM1_ESM.docx]

**Fig. S1.** The endoPG secretion of YKM1012 by cultivation in SD medium for 14 h under different temperatures staining by CBB.


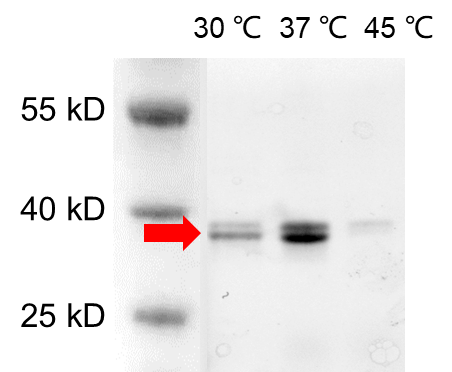


**Fig. S2.** Suppression of endoPG activity in engineered strain (YKM1013) under higher temperature. The enzyme activity assay was conducted by the volume ratio of 1:4 (0.2 mg/mL endoPG and 1% pectin) at 55 ℃ for 1 h. The YKM1013 was cultivated upon SD medium at varied incubation durations. The endoPG derived from YKM1013 cultivated at 30 ℃ for 10 h was performed as the control group.

**
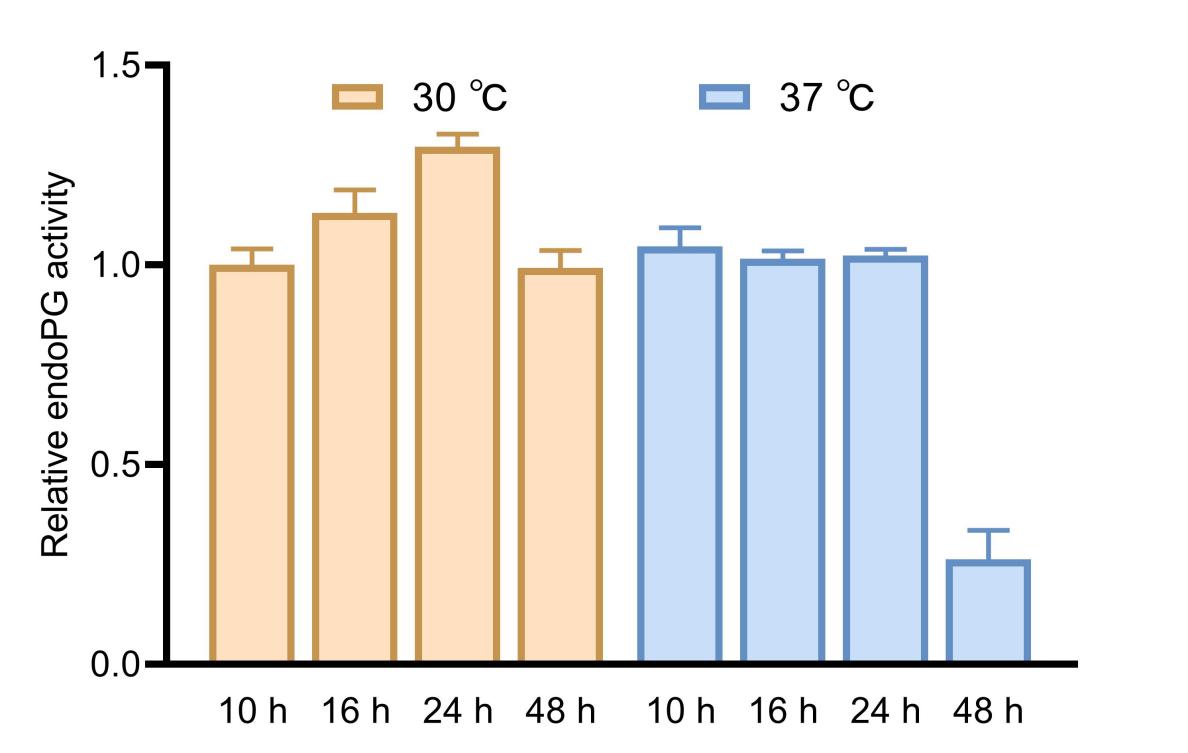
**

**Fig. S3.** Hydrolysis activity of endoPG derived from engineered strain (YKM1013) by incubating at varied temperatures for 1 h. The enzyme activity assay was conducted by the volume ratio of 1:4 (0.2 mg/mL endoPG and 1% pectin). The YKM1013 was cultivated upon SD medium at 30 ℃ for 24 h. The sample without endoPG was used as the blank control, and their values were subtracted from the experimental groups for the calculation of reducing sugar.

**
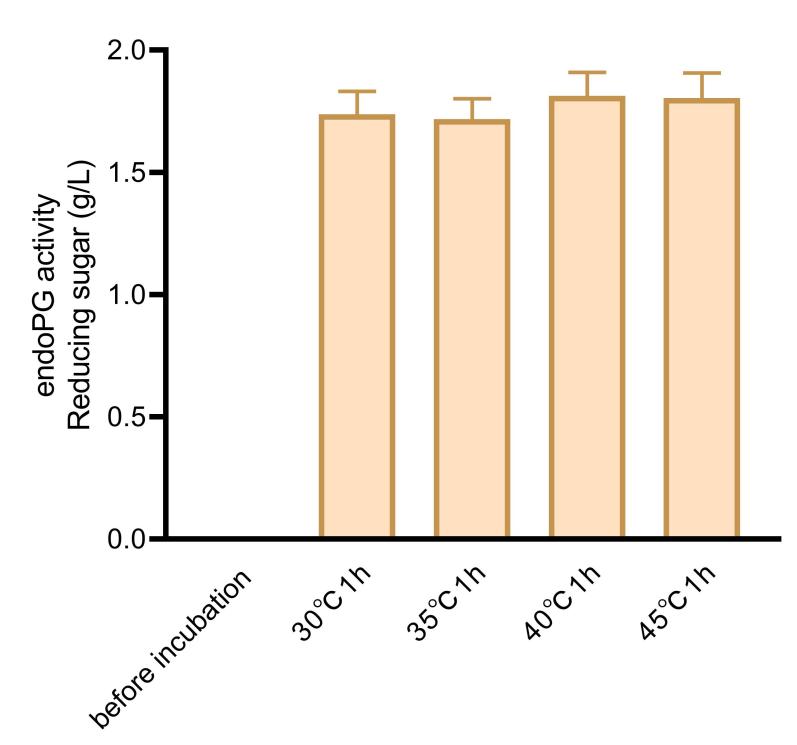
**

**Fig. S4.** Thermal stability of endoPG derived from engineered strain (YKM1013) by the volume ratio of 1:4 (0.2 mg/mL endoPG and 1% pectin) at 45 ℃ for 30 min through prolonged pre-treatment durations. The YKM1013 was cultivated in SD medium at 30 ℃ for 24 h. The sample without pre-treatment was used as the blank control.

**
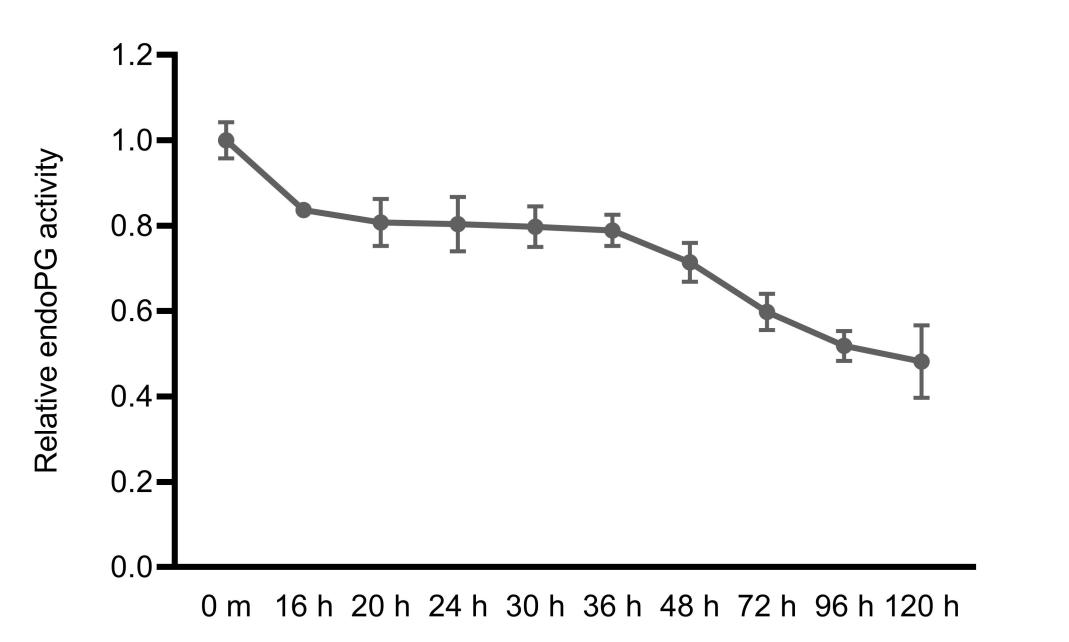
**

**Fig. S5.** The endoPG activity derived from engineered strain (YKM1013) in sodium acetate buffer (pH 5.5) by the volume ratio of 1:9 (0.6 mg/mL endoPG and 1% pectin) at 45 ℃ at varied durations. The YKM1013 was cultivated upon SD medium at 30 ℃ for 24 h. The endoPG and 1% pectin were dissolved in sodium acetate buffer (pH 5.5).

**
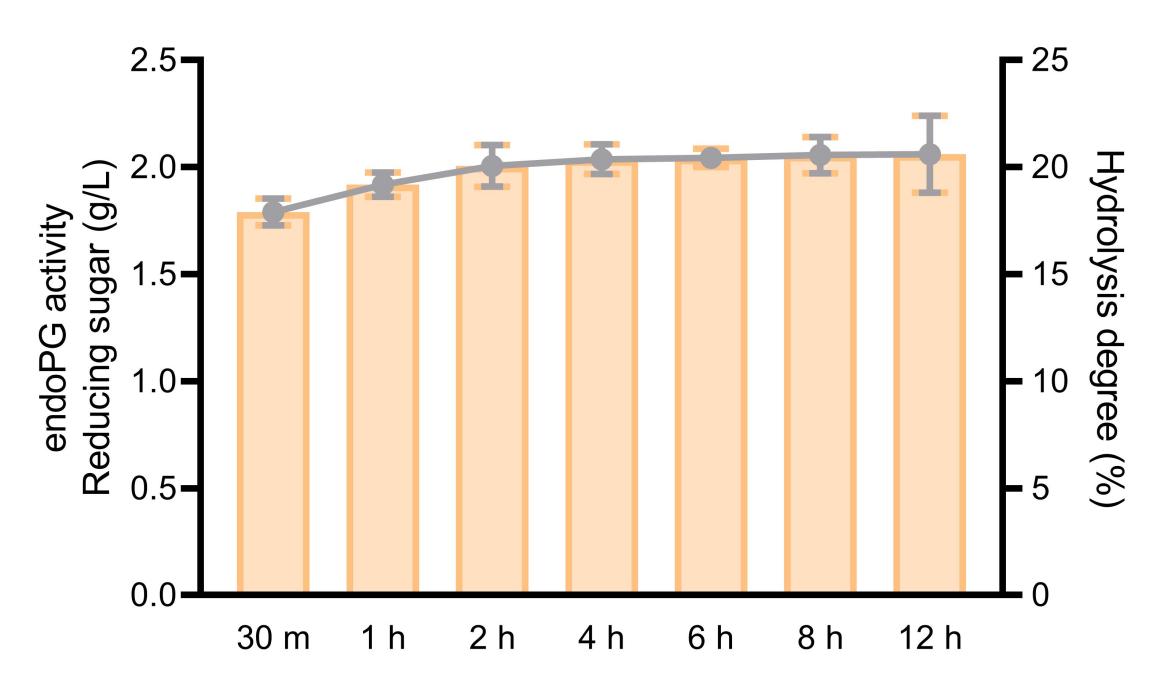
**

Left Y-axis, Reducing sugar concentration; Right Y-axis, Hydrolysis degree.

**Fig. S6.** The chromatograms for LC-MS/MS detection of YKM1012 and YKM1015.


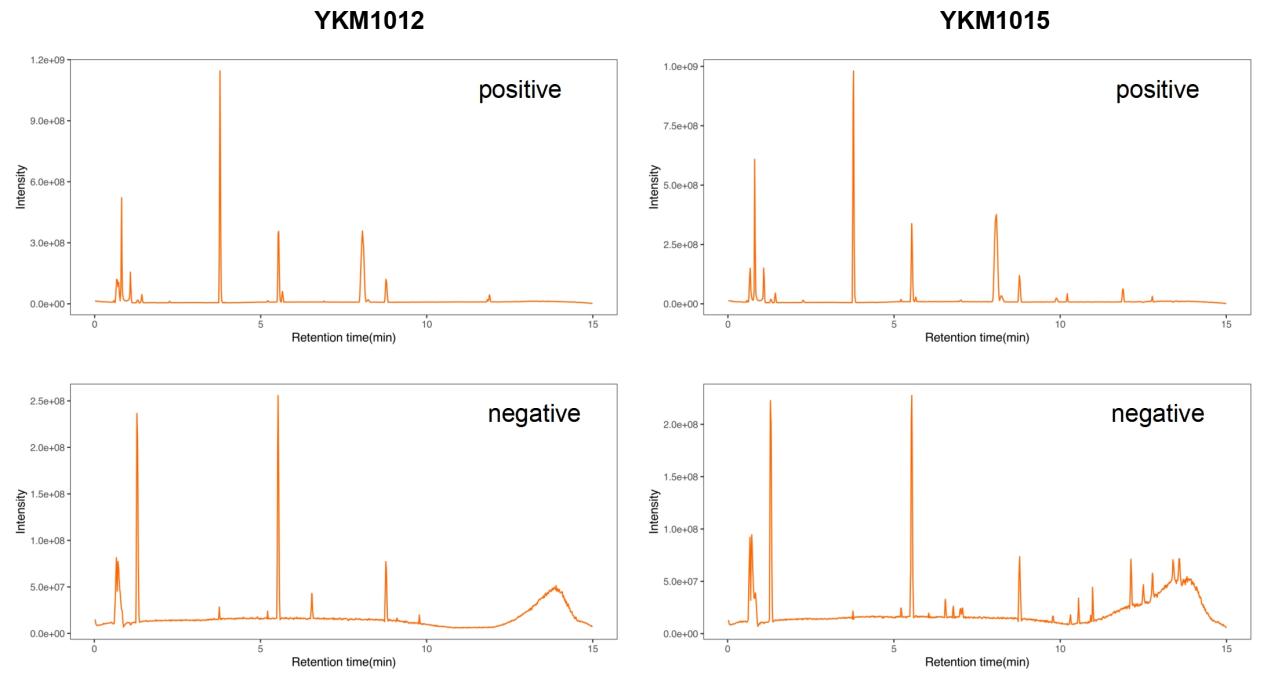


**Fig. S7.** The comparison of metabolic profile between YKM1012 and YKM1016 strains.


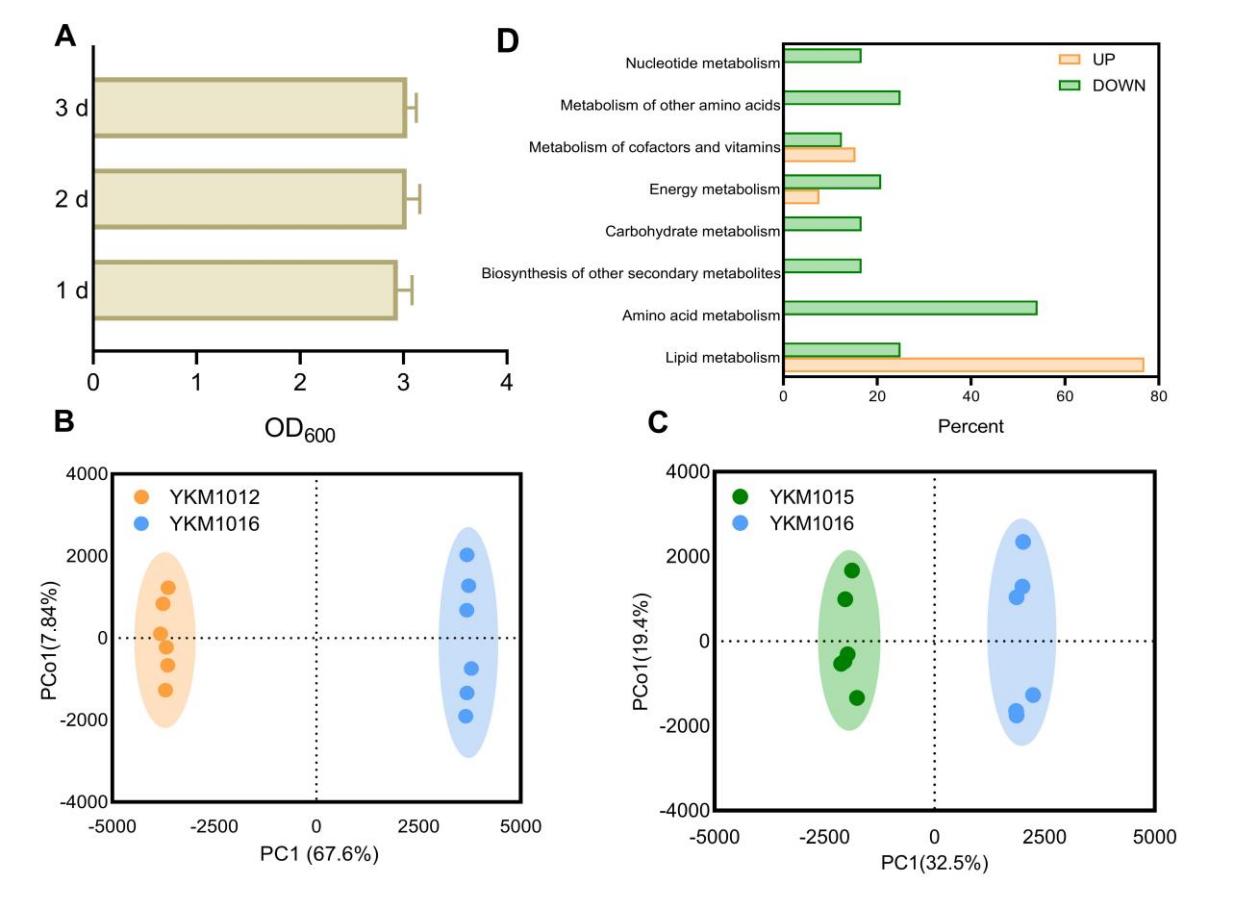


(A) The cell growth of engineered strain (YKM1016) by cultivation in pectin hydrolysate. (B) OPLS-DA load diagram of differential metabolites between YKM1012 and YKM1016 strains, and (C) between YKM1015 and YKM1016 strains. (D) The summary of classified metabolism pathway of significantly up- and down-regulated metabolites between YKM1012 and YKM1016 strains. *light orange* represented the up-regulated metabolism pathway and *light green* represented the down-regulated metabolism pathway.

**Fig. S8.** The metabolites with significant differences in pathway enrichment between YKM1012 and YKM1015 strains by cultivation in SD medium (2% glucose) at 30 ℃ for 72 h.

**
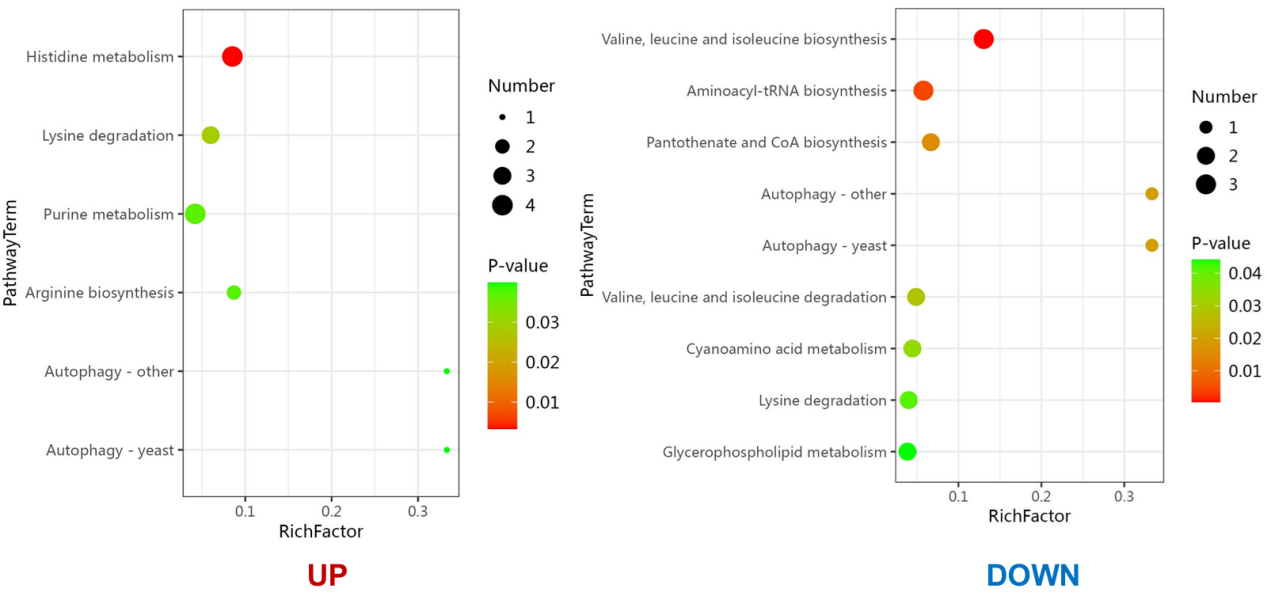
**

Table S1 The summary of plasmids used in this study

| **Plasmids name** | | **Plasmids description** | **References** |
| --- | --- | --- | --- |
| pIW1146 | 700bp LYS1 up and downstream | | Li et al. 2021 |
| pIW1136 | 700bp XYL2 up and downstream | | Li et al. 2021 |
| pIW1149 | 700bp SDL1 up and downstream | | Li et al. 2021 |
| pIW1135 | 700bp ABZ1 up and downstream | | Li et al. 2021 |
| pIW601 | P*_scTEF1_*-KmCas9-SV40-*ScCYC1t*, P*_KmPRP1_*-tRNA^Gly^-PspXI recognition site-SUP4 | | Löbs et al. 2017;  Li et al. 2021 |
| pIW1137 | pIW601 with LYS1 targeting sgRNA | | Li et al. 2021 |
| pIW447 | pIW601 with XYL2 targeting sgRNA | | Li et al. 2021 |
| pIW1132 | pIW601 with SDL1 targeting sgRNA | | Li et al. 2021 |
| pIW1134 | pIW601 with ABZ1 targeting sgRNA | | Li et al. 2021 |
| PGko-sg | pIW601 with PGU1 targeting sgRNA | | This study |
| 1135-PGU1 | Replacing the EGFP in pIW1135 with KmPGU1 | | This study |
| 1146-GAAC | Replacing the EGFP in pIW1146 with AnGAAC | | This study |
| 1136-GAR1 | Replacing the EGFP in pIW1136 with TrGAR1 | | This study |
| 1136-GatA | Replacing the EGFP in pIW1136 with AnGatA | | This study |
| 1149-GAR1 | Replacing the EGFP in pIW1149 with TrGAR1 | | This study |
| 1149-GatA | Replacing the EGFP in pIW1149 with AnGatA | | This study |
| 1135-LGD1 | Replacing the EGFP in pIW1135 with TrLGD1 | | This study |
| 1135-V-LGD1 | Replacing the EGFP in pIW1135 with V-TrLGD1 | | This study |
| PGko-HD | 700bp PGU1 up and downstream | | This study |

Table S2 Gene sequences in this study

| ***TrGAR1*** |
| --- |
| ATGGTTGCTACTTCTTTTAAACTCAACAACGGTTTGGAGATACCTGCTGTCGGATTAGGCACTTGGCAATCGAAGGCTGGCGAAGTTAAGGCCGCCGTTTCTTATGCCCTTCAGATTGGTTACAAGTTAATAGATGGTGCATACTGCTATGGTAATGAAGATGAAGTTGGAGAAGGGTTGAAAGAAGCTTTTGCGGCTGGGGTCAAAAGAGAGGACATTTTCGTTGTGACTAAGATATGGGCTACTTATAACACAAGGGTGGTGTTGGGTTTGGATAAGTCTTTGCGGAGTTTAGGTTTAGATTATGTTGATCTGTTATTGGTTCACTGGCCAGTGTTGCTCAATCCTGAAGGTAATCATGACAAGTTTCCTACTCTTCCTGACGGTAAGAGAGATGTGATATGGGACTACAATCACGTCGATGGATGGAAGCAAATGGAAGCTGTACTTGCGACTGGGAAGACAAAATCCATAGGTGTTTCTAACTATTCCAAAAAGTATCTTGAGCAACTTTTGCCACATGCAACGGTTATTCCTGCTGTCAACCAAATCGAAAATCATCCTTCATTACCACAACAGGAGATTGTTGATTTTTGCAAAGAAAAGGGAATCCATATTATGGCCTATTCCCCATTAGGCTCAACTGGATCTCCATTGATGTCCGCCGATCCAGTAGTGAAGATCGCTGAAAAAAAAGGAATTAGCCCGACTACTGTGCTGCTGAGCTATCACGTAAACAGAGGGTCCACGGTGTTGGCCAAGTCAGTAACGCCTGCACGAATTAAAGCCAACTTAGAGATCGTCGACCTCGATGACGAAGATATGAAGTTGTTAAATGACTACTCAAACGACCTCGCATCCAAGGGTGAATTGAAAAGGTATGTTTATCCACCCTTCGGAATTGACTTTGGGTTCCCAGACAAAAGTGGATCCTAAG |
| ***V-TrLGD1*** |
| ATGTCTAAAGGTGAAGAATTATTCACTGGTGTTGTCCCAATTTTGGTTGAATTAGATGGTGATGTTAATGGTCACAAATTTTCTGTCTCCGGTGAAGGTGAAGGTGATGCTACTTACGGTAAATTGACCTTAAAATTGATTTGTACTACTGGTAAATTGCCAGTTCCATGGCCAACCTTAGTCACTACTTTAGGTTATGGTTTGCAATGTTTTGCTAGATACCCAGATCATATGAAACAACATGACTTTTTCAAGTCTGCCATGCCAGAAGGTTATGTTCAAGAAAGAACTATTTTTTTCAAAGATGACGGTAACTACAAGACCAGAGCTGAAGTCAAGTTTGAAGGTGATACCTTAGTTAATAGAATCGAATTAAAAGGTATTGATTTTAAAGAAGGTGGTAACATTTTAGGTCACAAATTGGAATACAACTATAACTCTCACAATGTTTACATCACTGCTGACAAACAAAAGAATGGTATCAAAGCTAACTTCAAAATTAGACACAACATTGAAGATGGTGGTGTTCAATTAGCTGACCATTATCAACAAAATACTCCAATTGGTGATGGTCCAGTCTTGTTACCAGACAACCATTACTTATCCTATCAATCTGCCTTATCCAAAGATCCAAACGAAAAGAGAGATCACATGGTCTTGTTAGAATTTGTTACTGCTGCTGGTATTACCCATGGTATGGATGAATTGTACAAAGGTAGCAGCGAGGTTACCATAACTGGATTTCGTTCCAGAGATGTGCGATTCCCGACATCCCTTGATAAAACTGGGTCTGATGCCATGAATGCAGCAGGGGACTATTCTGCTGCTTATTGTATACTCGAAACTGACAGTGCTCACAGTGGCCACGGCATGACTTTCACGATAGGTAGAGGGAACGATATTGTATGTGCTGCAATTAATCACGTTGCTGACCGTCTGAAAGGAAAAAAATTGTCTAGTTTGGTCGCTGATTGGGGTAAGACTTGGCGTTACCTGGTTAACGACAGTCAATTAAGATGGATTGGTCCAGAAAAGGGCGTTATACATCTCGCGCTGGGTGCAGTAGTCAATGCAGTATGGGATCTCTGGGCTAAGACGTTGAATAAACCAGTTTGGAGAATTGTGGCAGATATGACGCCAGAGGAGTATGTTAGGTGTATCGACTTTAGATACATAACAGATGCCATTACTCCTGAGGAGGCTGTCGCAATGCTGAGAGAGCAAGAGGCTGGAAAGGCAAAAAGGATTGAGGAAGCTTTACAGAATCGTGCCGTCCCAGCATATACTACAAGTGCGGGTTGGTTAGGCTACGGTGAAGACAAGATGAAACAGTTGCTAAGAGAAACGTTGGCTGCTGGTTATAGACACTTTAAGGTTAAAGTAGGCGGTTCTGTTGAAGAAGATAGGCGTAGGTTGGGTATCGCAAGGGAAATTTTGGGCTTCGACAAAGGTAACGTGTTGATGGTCGATGCAAATCAAGTCTGGAGTGTACCAGAGGCTATTGATTATATGAAACAGCTCAGCGAGTATAAACCTTGGTTCATAGAGGAACCTACAAGTCCCGATGACATAATGGGCCATAAAGCAATTAGGGATGCTCTTAAACCCTATGGTATTGGAGTTGCTACCGGGGAGATGTGTCAGAATAGAGTAATGTTCAAGCAGTTGATTATGACAGGTGCAATAGATATTTGCCAAATTGACGCCTGTAGACTAGGTGGCGTCAACGAAGTGCTGGCCGTCCTATTGATGGCAAAGAAATACGGAGTTCCAATAGTTCCCCACTCTGGCGGTGTTGGACTACCGGAGTATACACAACACTTATCTACAATCGATTACGTTGTGGTTTCTGGCAAATTGTCGGTCTTAGAGTTCGTGGATCACTTACATGAGCATTTTCTCCATCCATCTGTAATCAAGGATGGGTATTATCAAACACCAACCGAGGCTGGATATTCAGTGGAAATGAAACCCGAGTCTATGGACAAATATGAGTACCCTGGTAAGAAAGGTGTTTCTTGGTGGACCACCGATGAAGCTTTACCAATTTTAAACGGCGAAAAAATAGGATCCTAAG |
| ***TrLGD1*** |
| ATGAGCGAGGTTACCATAACTGGATTTCGTTCCAGAGATGTGCGATTCCCGACATCCCTTGATAAAACTGGGTCTGATGCCATGAATGCAGCAGGGGACTATTCTGCTGCTTATTGTATACTCGAAACTGACAGTGCTCACAGTGGCCACGGCATGACTTTCACGATAGGTAGAGGGAACGATATTGTATGTGCTGCAATTAATCACGTTGCTGACCGTCTGAAAGGAAAAAAATTGTCTAGTTTGGTCGCTGATTGGGGTAAGACTTGGCGTTACCTGGTTAACGACAGTCAATTAAGATGGATTGGTCCAGAAAAGGGCGTTATACATCTCGCGCTGGGTGCAGTAGTCAATGCAGTATGGGATCTCTGGGCTAAGACGTTGAATAAACCAGTTTGGAGAATTGTGGCAGATATGACGCCAGAGGAGTATGTTAGGTGTATCGACTTTAGATACATAACAGATGCCATTACTCCTGAGGAGGCTGTCGCAATGCTGAGAGAGCAAGAGGCTGGAAAGGCAAAAAGGATTGAGGAAGCTTTACAGAATCGTGCCGTCCCAGCATATACTACAAGTGCGGGTTGGTTAGGCTACGGTGAAGACAAGATGAAACAGTTGCTAAGAGAAACGTTGGCTGCTGGTTATAGACACTTTAAGGTTAAAGTAGGCGGTTCTGTTGAAGAAGATAGGCGTAGGTTGGGTATCGCAAGGGAAATTTTGGGCTTCGACAAAGGTAACGTGTTGATGGTCGATGCAAATCAAGTCTGGAGTGTACCAGAGGCTATTGATTATATGAAACAGCTCAGCGAGTATAAACCTTGGTTCATAGAGGAACCTACAAGTCCCGATGACATAATGGGCCATAAAGCAATTAGGGATGCTCTTAAACCCTATGGTATTGGAGTTGCTACCGGGGAGATGTGTCAGAATAGAGTAATGTTCAAGCAGTTGATTATGACAGGTGCAATAGATATTTGCCAAATTGACGCCTGTAGACTAGGTGGCGTCAACGAAGTGCTGGCCGTCCTATTGATGGCAAAGAAATACGGAGTTCCAATAGTTCCCCACTCTGGCGGTGTTGGACTACCGGAGTATACACAACACTTATCTACAATCGATTACGTTGTGGTTTCTGGCAAATTGTCGGTCTTAGAGTTCGTGGATCACTTACATGAGCATTTTCTCCATCCATCTGTAATCAAGGATGGGTATTATCAAACACCAACCGAGGCTGGATATTCAGTGGAAATGAAACCCGAGTCTATGGACAAATATGAGTACCCTGGTAAGAAAGGTGTTTCTTGGTGGACCACCGATGAAGCTTTACCAATTTTAAACGGCGAAAAAATATAA |
| ***AnGAAC*** |
| ATGCCTTTCACACCATTAAGGCCAGGCGTGTATGCTCCAACCATGACCTTTTTTGACCCCTCTACAGAGGATCTTGATGTCCCGACGATTAGAAAGCACGCGGTCAGGCTTGCTAAAGCAGGGTTGGTCGGTCTGGTTTGCATGGGTTCCAACGGTGAAGCAGTGCATCTGACGAGAGCTGAAAGAAAAACCGTTATCAATGAAACCAGAAGTGCCTTGGTTGAAGCTGGGTTTTCAAACGTACCAGTGATTGCAGGTGCTTCCGAACAATCAATTAGGGGCACGATAGAACTTTGCAAGGAATCTTACGAAGCAGGTGCGGAGTACGCTCTAATTGTACCGCCTTCCTATTATAGGTACGCAACAGGCAACGACCAAACATTATACGAGTTTTTTACATCAGTGGCAGATGGTTCACCAATACCACTGATCCTATACAATTACCCTGGCGCAGTGGCTGGTATTGACATGGATAGCGACCTTATTATTCGTATTAGCCAACATCCAAACATTGTTGGAACTAAGTTCACCTGTGCTAATACTGGTAAACTGACTAGAGTGGCATCAGCTCTCCACGCTATCACACCCCCTTCCCCACTGGCCCCTGCTCAACGTAAATTTCCATCAACAAAGACTGAGGCAAATCACCCGTACGTAGCCTTCGGTGGGATAGCTGACTTCTCTTTACAAACATTAGCTTCTGGTGGCAGCGCAATTCTGGCTGGTGGTGCTAATGTTATACCTAAGTTGTGTGTACAAATTTTTAATTTGTGGTCAGCTGGTCGCTTTACTGAAGCTATGGAAGCTCAGGAATTACTCTCCAGAGCAGATTGGGTATTAACCAAAGCTGCCATTCCTGGTACAAAATCAGCCATTCAATCATATTACGGGTACGGAGGGTTTCCCAGAAGACCGTTAGCTAGATTAAGTGCAGAACAAGCTGAGGCTGTCGCCGAGAAGATAAAGGATGCCATGGAGGTTGAAAAAAGTCTACCTGACATCGCAGGATCCTAAG |
| ***AnGatA*** |
| ATGAGTCTCCTCAAGAACTATCGTGTTTACCTCCTCACAGCAGTGGCCTACTCGGGGTCACTTCTTTTCGGCTATGATACGGGTGTTATGGGAAGTGTTCTTTCCCTGACCAGCTTCAAGGAAGATTTTGGGATACCTACTGGCTCTTCTGGTTTTGCGTCCTCCAAGAGCTCCGAAATCTCATCCAATGTCGTATCCTTGCTGACTGCGGGCTGCTTCTTCGGCGCCATCTTCGCTGCCCCCCTTAACGAGCGCATTGGCCGACGCTATGCCCTGATGATCTTCACCGTCATATTTCTCATTGGAGCAGCTGTTCAAGTTGCATCGAAGCATCACATTGGACAGATCTATGGTGGCCGTGTCATAGCGGGCTTGGGAATTGGTGGAATGTCCAGTATCACGCCTGTGTTCGTGAGCGAGAACTGCCCCCCAAGCATTCGAGGCCGAGTTGCTGGAATGTTCCAGGAGTTCCTGGTCATTGGAAGCACCTTTGCCTATTGGCTGGACTATGGTGTCTCCCTGCACATTCCCTCAAGCACGAAGCAATGGCGTGTCCCAGTCGCTGTCCAGCTTATTCCTGGCGGTCTTATGCTACTCGGCCTTTTCTTCCTCAAGGAGTCTCCTCGTTGGCTGGCTGGCAAAGGACGCCATGAAGAAGCTCTTCAGTCACTCGCATACATCAGGAACGAGTCCCCTGATAGCGAGGAGATTCAGAAAGAGTTTGCTGAGATCCGAGCAGCCATCGATGAAGAGGTAGCAGCCACCGAAGGGCTGACTTACAAAGAGTTTATCCAGCCGAGTAACCTCAAACGCTTCGGTTTCGCCTTCACTCTCATGCTTTCACAACAATTCACGGGTACCAATTCGATCGGTTACTACGCTCCTGAGATCTTCCAAACTATCGGGCTTAGCGCGACTAACTCGTCTCTTTTTGCTACTGGCGTCTACGGAACTGTGAAGGTTGTCGCGACGGCTATCTTCCTATTTGTTGGTATCGACCGTTGGGGCCGTAAGCTCAGTCTGGTTGGCGGATCCATTTGGATGGCCAGCATGATGTTCATCATTGGCGCTGTATTGGCTACGCACCCGCCCGACACAAGCGCCAGTGGCGTCTCCCAGGCTTCTATCGCCATGGTGGTCATGATCTATCTCTACGTGATCGGCTATTCGGCTTCATGGGGCCCTACCCCCTGGGTGTATGTGAGCGAGATCTTCCCGACCCGGCTACGTTCATACGGTGTCGGTCTGGCAGCCACATCCCAGTGGCTCTGGAGTTTCGTCGTCACTGAAATCACCCCCAAAGCCGTCCACAACATCGGCTGGCGCACATTCCTCATGTTTGGTATCTTCTGCGTTGCCATGTGTGTCTTCGTCATTGTCTTCGCCAAGGAAACCAAGGGCCGCAGTCTTGAGGATATGGACATCCTCTTCGGTGCTGTCAATGAGGCCGACCGCCGTGCTGCGGTGGAACATACCATGCACAAGCGCGGCTCCTCTCACATCGAGGACGTGGATGAGGAAACCGAGCGTGTTCGCCATGAGCAGGACAAGGTCTAG |
| ***KmPGU1*** |
| ATGCATGTAGATAGCAGCAACGACAACAGTAACGACAATAACGATACCAACAAACAACAGCAACAGAAAGTAAGCTCAGAAACCAAAAAAAAAAAAATGTTATTCAGCAACACCTTATTAATCGCAGCAGCTAGTGCATTATTAGCTGAAGCTTCTCCATTGGAAAAGAGAGACAGTTGTACCTTGAGTGGGAAGACAGCAGGAGGTGGATTGTCCAACTGTGCCACGGTCACTGTCAACAACGTTGAAGTCCCAGCTGGTAAGACCTTGGACTTGACAGGCTTGCAAGACGGTGCGACAGTTAATTTCGTCGGGCAGGTTACCTTTGATTACGATGAATGGGTGGGTCCATTGGTCTCCATCTCCGGTAAGAACATCAAGGTGGTGGGTAAGTCTGGCCACTTGTTAGATGGTGATGGTGCACGTTGGTGGGACGGGAAGGGTGACAGTGGTAAAAAGGTGAAGCCTAAGTTCATGAGCTTGAAATTGACTGGCAACTCAGATGTCGGTGGGTTGCAAATCAAGAATACCCCAATTCAAGCTATCTCAGTGAACTCTTGTAGTGACACTGTGATTCACGATGTCACCATTGACAACAGTGATGGTGACAAGGACAGCTTGGGTCACAACACTGATGGTTTCGATGTTGGTAACGTTAACAACGTCACCATTGAAAACTGTCATGTCTACAACCAAGACGACTGTATCGCCGTCAACTCCGGTACCGGTGTCTACTTCAAGAACAACTACTGTTCTGGTGGTCATGGTGCTTCCATTGGTTCAGTCGGTCTTCGCTCAAACAATGTGGTTGACACCGTTTACTTCGAGAACAACCAAATTGTCAACTCTGACAACGGTTTGAGAATTAAGACCATTCAAAAGGCCACTGGTTCCGTTAACAACGTGCACTTCTTGTCCAACACTATCTCCGGCATCAGAAAGTTCGGTATTGTTGTTGAAACTGATTACAGCAGTGGATCCACCACCGGTACCCCAGGTAGCAAGGTCCCAATCACCAACTTCGAAGTCGATGGTTTGACTGGTTCAGTTGACTCTTCCGCTTACAGAGTCAAGATCTTGGTTGCTGGTGCTTCTAAGTGGACTTGGAAGGATGTTGATATCACTGGTGGTTCTTCTTTCGGTTCATGTACTGGTATTCCATCTGGTAGCGGAGCTTCCTGTTAA |

Table S3 Primers for cloning and gene sequence verification in this study

| **Gene cloning** |  |
| --- | --- |
| GAR1-clone-F | CCCCCGGGATGGTTGCTACTTCTTTTAAA |
| GAR1-clone-R | TAATTACATGACTCGAGCTTAGGATCC |
| GAAC-clone-F | ATCCCCCGGGATGCCTTTCACACCATTAA |
| GAAC-clone-R | ACATGACTCGAGCTTAGGATCCTGCGATGT |
| LGD1-clone-F | ATCCCCCGGGATGAGCGAGGTTACCATAA |
| LGD1-clone-R | CATGACTCGAGTTATATTTTTTCGCCG |
| VLGD1-clone-F1 | TAGAACTAGTGGATCCCCCGGGATGTCTAAA |
| VLGD1-clone-R1 | TTATGGTAACCTCGCTGCTACCTTTGTACAATTCATCCATACC |
| VLGD1-clone-F2 | GGTATGGATGAATTGTACAAAGGTAGCAGCGAGGTTACCATAA |
| VLGD1-clone-R2 | CATAACTAATTACATGACTCGAGCTTAGGATCCTATTTTTTCGCCG |
| AnGatA-clone-Fp | TTCTAGAACTAGTGGATCCCCCGGGATGAGTCTCCTCAA |
| AnGatA-clone-Rp | GACATAACTAATTACATGACTCGAGCTAGACCTTGTCCTGC |
| PGU1-clone-Fp | CTAGAACTAGTGGATCCCCCGGGATGCATGTAGATAGCAGCAAC |
| PGU1-clone-Rp | CATAACTAATTACATGACTCGAGTTAACAGGAAGCTCCGCTAC |
| PGU1-HD-Fp1 | GGGCGAATTGGAGCTCCACCGCGGTGGCATCGCCGATCTCAAGTGT |
| PGU1-HD-Rp1 | CTGTCCACCAGTCATGCTAGCGAGCTTACTTTCTGTTGCTGTT |
| PGU1-HD-Fp2 | AACAGCAACAGAAAGTAAGCTCGCTAGCATGACTGGTGGACAG |
| PGU1-HD-Rp2 | GGGAACAAAAGCTGGTACCGGCCGGACGAGGTCTATCAAACCTAC |
| PGU1-sg-Fp | GAATCCCGTCAGTGTCAACcGATTGGGACCTTGCTACCTGgGTTTTAGAGCTAGAAATAG |
| PGU1-sg-Rp | CTATTTCTAGCTCTAAAACcCAGGTAGCAAGGTCCCAATCgGTTGACACTGACGGGATTC |
| **Sequence verification** |  |
| LYS1-HD-clone-Fp | TTACAAGAACCAGGCTGGTTGGG |
| LYS1-HD-clone-Rp | CGACGGTAGTGTCCAATTTTGG |
| SDL1-HD-clone-Fp | CTAACGTTTCCGTAGACATTGTCG |
| SDL1-HD-clone-Rp | GCAATGTCAGTTAATTGGCTCCTAG |
| XYL2-HD-clone-Fp | CGCATGGTAGGCTAGATTTCGG |
| XYL2-HD-clone-Rp | GACAACTTCATTACCGTGATCTCTG |
| ABZ1-HD-clone-Fp | CTTCCTCAAGGCATTTCACACACACT |
| ABZ1-HD-clone-Rp | AAACGTCTTCAGGAAGCTCCATAGGC |

**References**

Li M, Lang XY, Moran Cabrera M, De Keyser S, Sun X, Da Silva N, Wheeldon I (2021) CRISPR-mediated multigene integration enables Shikimate pathway refactoring for enhanced 2-phenylethanol biosynthesis in *Kluyveromyces marxianus*. Biotechnol Biofuels 14(1):3.

Löbs AK, Engel R, Schwartz C, Flores A, Wheeldon I (2017) CRISPR-Cas9-enabled genetic disruptions for understanding ethanol and ethyl acetate biosynthesis in *Kluyveromyces marxianus*. Biotechnol Biofuels 10:164.
